# In Situ Assembly of Hydrogen‐Bonded Organic Framework on Metal–Organic Framework: An Effective Strategy for Constructing Core–Shell Hybrid Photocatalyst

**DOI:** 10.1002/advs.202204036

**Published:** 2022-10-18

**Authors:** Jianli Wang, Yifan Mao, Runze Zhang, Yanli Zeng, Changsheng Li, Bingjie Zhang, Jianhui Zhu, Jiawen Ji, Desheng Liu, Rumin Gao, Yongqiang Ma

**Affiliations:** ^1^ Department of Applied Chemistry College of Science China Agricultural University Beijing 100193 P. R. China; ^2^ Department of Chemistry University of Virginia Charlottesville VA VA 22094 USA; ^3^ Department of Engineering Systems and Environment University of Virginia Charlottesville VA VA 22904 USA; ^4^ College of Chemistry and Materials Science Hebei Normal University Shijiazhuang 050024 P. R. China; ^5^ College of Agronomy and Biotechnology China Agricultural University Beijing 100193 P. R. China; ^6^ Present address: Yuanmingyuan West Road No.2 Haidian District Beijing 100193 P. R. China

**Keywords:** core–shell structure, hydrogen‐bonded organic frameworks, metal–organic frameworks, photocatalysis

## Abstract

The hydrogen‐bonded organic frameworks (HOFs) have rarely been considered for photocatalytic application, given their weak stability and low activity. One presumably effective strategy to improve the photocatalytic performance of the HOFs is to produce a core–shell composite by fabricating a particular nanostructure using stable HOFs. To this end, the surface‐functionalized metal–organic frameworks (MOFs) are used as the host matrix to support the in situ assembly and subsequent multisite growth of the stable HOFs. MOF@HOF eventually obtains core–shell hybrids, i.e., NH_2_‐UiO‐66@DAT‐HOF. This newly synthesized core–shell nanostructure exhibits excellent stability and superb photocatalytic performance. For example, in terms of tetracycline degradation, the optimal composite presents an apparent reaction rate constant of 60.7 and 7.6 times higher than its parent materials NH_2_‐UiO‐66 and DAT‐HOF. Such a pronounced enhancement in photocatalytic efficiency of the hybrid material is attributed to the broader visible‐light utilization range compared to its individual parent material as well as the efficient separation of charge carriers supported by the S‐scheme heterojunction. In addition, it is particularly notable that the photocatalytic efficiency of the yielded core–shell nanostructure can remain high after several‐cycle applications. This work provides a universal scheme for synthesizing the MOF@HOF core–shell hybrids.

## Graphical abstract

The MOF@HOF materials with excellent photocatalytic efficiency and reusability are developed. The significant improvement can be attributed to the peculiar structure of the DAT‐HOF shell coated on the metal–organic frameworks (MOFs) core, which not only extends the utilization range of the visible light but also improves the hole–electron pair's separation because of the S‐scheme heterojunction.

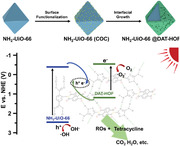

## 1 Introduction

The hydrogen‐bonded organic frameworks (HOFs), self‐assembled through hydrogen bonding, as a class of innovative crystalline porous materials have recently drawn significant attention owing to their well‐defined structures and versatility.^[^ ^1^ ^]^ Recently, the HOFs have been extensively investigated in photocatalysis, gas storage, sensing, and proton conduction.^[^ ^2^ ^]^ As for the area of photocatalysis, to improve the HOFs’ photocatalytic performance, many previous attempts have been applied. Metallization is a strategy to modify monomer of HOF. It is reported that metallizing the porphyrin centers could endow the HOFs with exhibit chemical stability, surface area, and catalytic activity,^[^ ^3^ ^]^ where the degree of metalation could affect the photocatalytic activity remarkably.^[^ ^4^ ^]^ Modifying HOF with metal (metal site postfunctionalization) is also effective for preparing robust functional HOFs to be a skeleton for supporting the photoactive units.^[^ ^5^ ^]^ Besides, loading metal or metal oxide onto the HOF photocatalysts is a potential method, which helps lead out the hot‐electron to construct photochemical stability of HOFs or fabricate heterostructure to reduce the recombination of charge carrier.^[^ ^6^ ^]^ In general, introducing metal into HOF is a common strategy for enhancing the photocatalytic performance of HOF‐based photocatalyst. However, applying these metal‐modified HOFs directly in pollutants’ photocatalysis would sometimes be inappropriate and often restrained by the instability of the decorated metal portion. Thus, we are seeking to find proper strategies to construct HOF‐based photocatalyst to simultaneously achieve strong photostability and high photocatalytic activity.

As an alternative, the development of hybrid composites like the core–shell materials has been verified to be effective in overcoming the photocatalytic defect of the HOFs. Such a merge process is expected to not only preserve the parental structural feature but also acquire enhanced performances compared to their parent components.^[^ ^7^ ^]^ Recently, a “bottle‐around‐ship” strategy was used to construct an up‐conversion nanoparticle core–shell nanostructure using perylene‐diimide‐based HOFs.^[^ ^8^ ^]^ The matched energy level between the up‐conversion nanoparticle core and the HOF shell endows core–shell nanostructure's excellent photothermal and photodynamic effects. However, that scheme only applies to specialized HOFs and is significantly constrained by the stabilizers' types. Plus, the fact that researches about fabricating HOF‐based core–shell structure are deficient^[^ ^3^ ^]^ and remain challenging, further exploration of more promising strategies dedicated to favorably forming HOFs‐included core–shell nanoparticles and thus effectively improving the HOFs’ photocatalysis is essential and critically important.

Metal–organic frameworks (MOFs), as a kind of crystalline porous materials, are characterized by adjustable and regular structures and considered suitable as the host matrix.^[^ ^9^ ^]^ For example, the MOF@COF (covalent organic framework) hybrids, synthesized through a sequential growth strategy, showed excellent efficiency in the one‐pot photocatalytic hydrogenation of nitrobenzene.^[^ ^10^ ^]^ Similarly, the MOF@MOF architectures exhibited high efficiency in the photocatalytic removal of Cr^6+^.^[^ ^11^ ^]^ The MOFs commonly display semiconductor characteristics, composed of photoactive ligands and metal units. They have great potential in photocatalysis because of their large specific surface area and much exposure to catalytic active sites.^[^ ^12^ ^]^

**Scheme 1:**
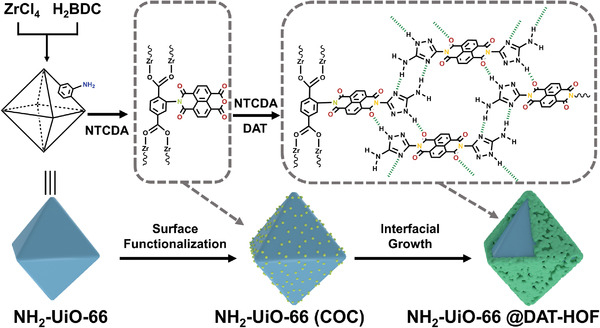
Illustration of the synthetic strategies for NH_2_‐UiO‐66@DAT‐HOF photocatalysts.

In light of these, the primary objective here is two‐fold, which includes whether the MOFs could serve as the host matrix to construct MOF@HOF hybrid materials and whether the photocatalytic performance of the newly formed nanostructure (MOF@HOF) is significantly strengthened than that of each parent material (MOF and HOF). As such, a typical Zr‐based MOF, NH_2_‐UiO‐66 (UiO), has been chosen for this work due to its high stability and structural support for consequent shell coating.^[^ ^9^ ^]^ The amino groups on the UiO permit further functionalization through covalently bonding at the molecular level. NH_2_‐UiO‐66@DAT‐HOF (U@H) core–shell hybrid materials were generated (**Scheme** **1**) through functionalization of the MOF core with naphthalenetetracarboxylic dianhydride (NTCDA), and the subsequent interfacial growth of the highly stable diamino triazole type multi‐site hydrogen bond HOF (DAT‐HOF) on the host.^[^ ^13^ ^]^ The resultant composites present structural synergy and significant improvement in the photocatalytic degradation of tetracycline (TC). Additionally, MOF@HOF materials behave with high structural and photochemical stability.

## 2 Results and Discussion

**Figure 1:**
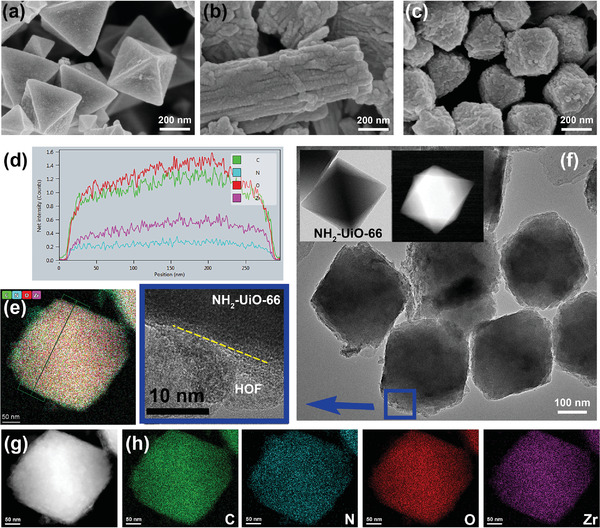
SEM images of a) UiO, b) HOF, c) U@H2 hybrid; d,e) EDX line profile of U@H2 hybrid; f) TEM and its enlarged images (blue framed) of U@H2 hybrid as well as the TEM and HADDF‐STEM images of UiO (inset); g,h) HAADF‐STEM and elemental mapping images of U@H2 hybrid.

The preparation process of the U@H hybrids is illustrated in Scheme 1. First, the UiO with octahedral morphology was synthesized through a solvothermal method (**Figure** **1**a; Figure S1, Supporting Information).^[^ ^14^ ^]^ Subsequently, the obtained UiO was further functionalized with NTCDA through the amidation reaction, producing anhydride‐modified NH_2_‐UiO‐66 (COC), i.e., N‐UiO. The morphology of the N‐UiO (Figure S2a, Supporting Information) still maintains octahedral and smooth, which is similar to that of pristine UiO, and the crystalline integrity of N‐UiO was confirmed by the X‐ray diffraction (XRD) analysis (Figure S3, Supporting Information). Moreover, the successful modification of the UiO with NTCDA was also identified by the asymmetric stretching of the imide ring at around 1691 cm^−1^, and other vibration features of the NTCDA in the Fourier transform infrared (FTIR) (Figure S4, Supporting Information).^[^ ^15^ ^]^ The covalent graft of the NTCDA could provide suitable sites for the self‐assembly of imide building blocks of the DAT‐HOF on the surface of the N‐UiO. Finally, the U@H hybrids were synthesized by dipping the N‐UiO in the precursor of the DAT‐HOF (DAT and NTCDA) at 170 °C for 6 h. By adjusting the concentrations of precursor, different U@H hybrids (referred to as the U@H1–4) were obtained.

Taking the U@H2 (2 times precursor of HOF for reacting) as an example, both the scanning electron microscopy (SEM) and transmission electron microscopy (TEM) reveal that the U@H2 hybrids keep octahedral after the in‐situ assembly growth of DAT‐HOF on UiO (Figure 1c,f). The enlarged image of Figure 1f clearly shows the interface layer where the rough DAT‐HOF shell is coated on the UiO core, where the thickness of shell layer is around 10–20 nm. The core–shell structure is also confirmed by the distinctly different high‐angle annular dark‐field scanning TEM images compared to UiO (Figure 1g; Figure S1c, Supporting Information). The energy dispersive X‐ray (EDX) line profile of the U@H2 hybrid (Figure 1d,e) shows that there were no obvious differences after the fabrication of core–shell structure. Plus, the coat of the DAT‐HOF shell on the UiO core is homogeneous (Figure 1h).

**Figure 2:**
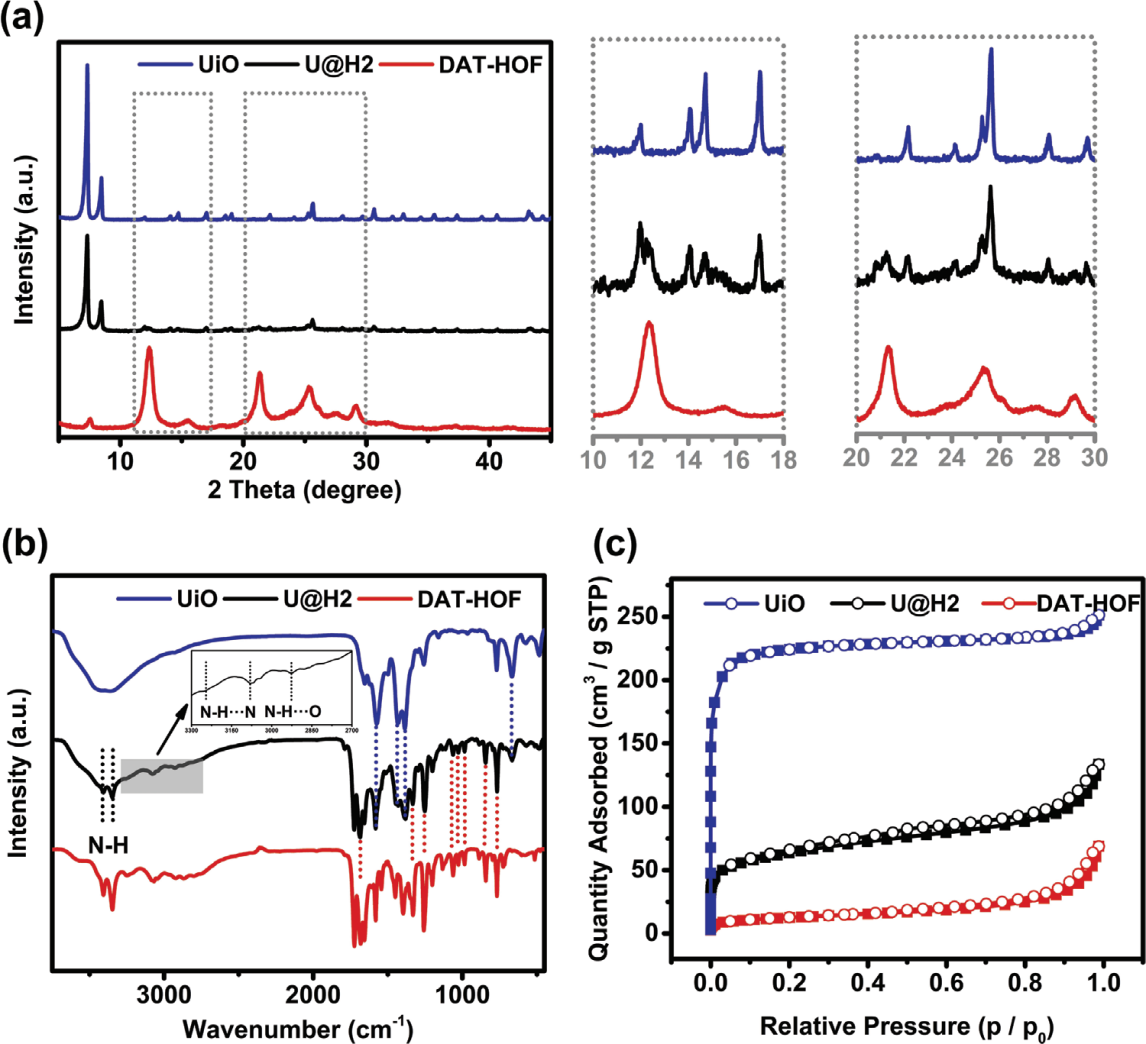
a) XRD pattern, b) FT‐IR spectrum, and c) N_2_ adsorption–desorption curves of UiO, DAT‐HOF, and U@H2 hybrid.

Furthermore, various characterization techniques have been adopted to identify the structural features of the U@H hybrids. The characteristic diffraction of the U@H hybrid in the XRD pattern maintains the same peaks as its parent UiO (**Figure** **2**a). Despite a relatively low content of the DAT‐HOF contained in the U@H composites, the weakened characteristic peaks of the DAT‐HOF at 12.3° and 21.4° (originated from the (001) and (210) planes of the DAT‐HOF)^[^ ^13^ ^]^ are still able to be distinguished in hybrids. With the increase in the concentration of the DAT and NTCDA, the characteristic diffraction peaks of DAT‐HOF in the U@H hybrid tend to become stronger (Figure S3, Supporting Information). The resulted FTIR spectrum of the U@H2 reveals both the characteristic absorption of UiO and DAT‐HOF (Figure 2b). The two peaks at around 3400 cm^−1^ are attributed to the terminal amino group in the triazole ring, while the hydrogen bonds (O···H–N and/or N···H–N) in the DAT‐HOF account for the signals centered at 3249, 3073, and 2922 cm^−1^. Additionally, the peak at 1410 cm^−1^ corresponds to the stretching vibration of imide, and the bands at around 1060 cm^−1^ are assigned to the in‐plane rocking vibration of the DAT‐HOF.^[^ ^13^ ^]^ Notably, as shown in Figure S5 (Supporting Information), the characteristic peaks of the UiO are also inherited by the newly produced U@H hybrids. Specifically, the typical peaks at 770 and 662 cm^−1^ are due to the absorption of the stretching mode of the Zr–O bond.^[^ ^16^ ^]^ The N–H scissors and C–C aromatic asymmetric stretch^[^ ^17^ ^]^ originated from UiO and could be responsible for the peaks of U@H composites at 1570, 1438, and 1386 cm^−1^. As the amount of the DAT‐HOF precursor increases, the peak corresponding to the stretching vibrations of the triazole ring situated at 843 cm^−1^ gets more prominent, whereas the Zr–O bond induced peak at 662 cm^−1^ declines likely because of the shielding effect caused by overly dense DAT‐HOF.

As illustrated in Figure S6 (Supporting Information), the peaks of the UiO and DAT‐HOF displayed in Raman spectra are overall in accordance with the previous reports,^[^ ^13^, ^17^ ^]^ even though some characteristic peaks of the UiO were indistinct because of the shell shielding of DAT‐HOF. In addition, the broadened peaks in the pattern of U@H2 indicate a strong interaction between the UiO and DAT‐HOF phases in the hybrid structure.^[^ ^18^ ^]^ In summary, the distinctive peaks of both parent products could be identified in the different types of spectra of the newly formed U@H composites, even if a few of them could be influenced by the concentrations of DAT‐HOF and thereby substantially drop.

The permanent porosities of samples were evaluated by the N_2_ adsorption–desorption experiments (Figure 2c). All UiO, DAT‐HOF, and U@H hybrids exhibit type I isotherm, demonstrating the microporous in samples. Moreover, the Brunauer–Emmett–Teller (BET) surface areas were calculated to be 43.94, 210.21, and 682.33 m^2^ g^−1^ for the DAT‐HOF, U@H2, and UiO, respectively. Apparently, the BET surface areas of the U@H hybrids are much smaller than that of the original UiO. The decreased BET surface areas of U@H hybrids were mainly due to the partial collapse of the framework and the blocking of the pore channel during the functionalization progress of yielding N‐UiO.^[^ ^15a^ ^]^ Furthermore, the stack of the DAT‐HOF layer can presumably further decrease the surface area (Figure S7b, Supporting Information). As shown in the thermal gravimetric analysis, the coated HOF shell inherits the high thermal stability of the DAT‐HOF thus the U@H2 exhibits similar weight loss curves with DAT‐HOF from thermal gravimetric analysis (Figure S8a, Supporting Information). The incomplete combustion might happen when HOF combust in the absence of oxygen (in N_2_ atmosphere). Once in the air atmosphere (Figure S8b, Supporting Information), HOF would be complete combusted at around 700 °C. The content of HOF in U@H2 could be calculated to be 19.8% by the thermogravimetric analysis (1–29.5%/36.8%).

**Figure 3:**
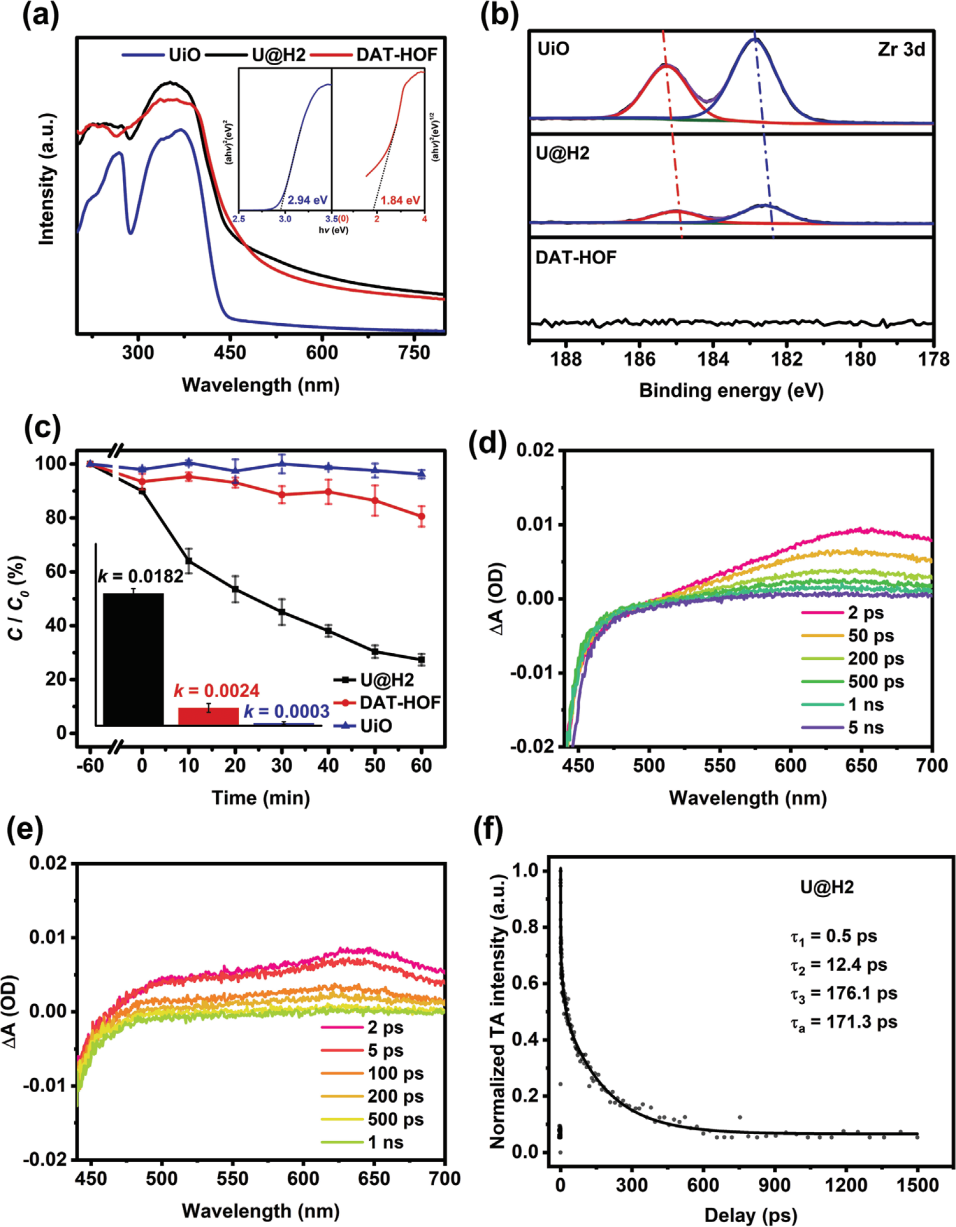
a) UV–vis DRS spectra with the inset of Tauc plots; b) high‐resolution Zr 3d XPS spectrum of the UiO, DAT‐HOF, and U@H2 hybrid; c) Photodegradation efficiencies of TC and the apparent reaction rate constants (inset) in the presence of different photocatalysts; TAS spectra of d) UiO and e) U@H2 following excitation by a 400 nm pump. f) Normalized time profiles of TAS at 650 nm.

Furthermore, the photochemical properties of the samples were separately investigated in detail. As shown in the solid‐state UV–vis diffuse reflectance spectroscopy (DRS) spectrum (**Figure** **3**a), the U@H2 shows a broad absorption span with a right edge at around 480 nm, which is similar to that of the DAT‐HOF while much larger than that of the UiO (425 nm). The extended absorption ranges covering from 400 to 800 nm signifies the excellent light absorption ability of the U@H2, demonstrating the synergistic effect resulting from the effective connection between the UiO and DAT‐HOF.^[^ ^10a^ ^]^

To verify the necessity of surface functionalization in constructing the core–shell structure, the comparative experiment (Experimental Section, Supporting Information) that produces the UiO/DAT‐HOF (U/H) aggregates by directly using pristine UiO and without any functionalization treatment was conducted. As a result, the core–shell structure that appears in the U@H hybrids cannot be observed in the U/H aggregates. Instead, the independent nucleation causes a random distribution of bulk DAT‐HOF around the UiO. Such a pronounced structural difference proves that surface functionalization of the host matrix is a prerequisite for the site‐selective assembly of the DAT‐HOF on the UiO surface. The incomplete core–shell structure in U/H composites leads to a much weaker synergistic effect, reflected by a decrease in the absorption edge and tail in the UV–vis DRS results (Figure S9, Supporting Information). Compared to the U@H2, the degradation of the light absorption ability of the U@H3 and U@H4 that formed using a larger amount of the DAT‐HOF are also attributed to the generation of the above‐mentioned U/H aggregated structure (Figure S2, Supporting Information). Since the functionalized groups on the UiO surface are deficient in guiding the assembly of all the available DAT‐HOF (in the cases of the U@H3 and U@H4, as shown in Figure S10 in the Supporting Information), excessive DAT‐HOF is inclined to independently grow and simply impose on the exterior layer of the UiO as the U/H composites, reducing their light absorption capability. Therefore, efficient construction of the U@H photocatalyst relies on the procedure of surface functionalization. As can be seen in the SEM images, when the precursor of HOF attends to a certain value, the bulk DAT‐HOF appears (U@H3). To get know the excessive precursor would affect the thickness of HOF shell, U@H1.5 and U@1.5 were prepared and all the TEM images of U@H hybrid particles were supplied in Figure S11 in the Supporting Information. On the whole, as the amount of precursor of HOF increases, the thickness of HOF shell gets raised. When the additive gets 2.5, 3, and 4 times of the precursor of HOF, the changes of thickness are small and the thickness remained around 18 nm (U@H2.5, U@H3, and U@H4). Significantly, when excessive precursor of HOF was reacted, the thickness of HOF layer had a few changes when reach a certain value. Therefore, the excessive precursor may not be useful to the extending of HOF shell but the independent nucleation of bulk DAT‐HOF. Consequently, the addition of an appropriate volume of the DAT‐HOF is also responsible for the efficient construction of the U@H photocatalyst.

The X‐ray photoelectron spectroscopy (XPS) technique was carried out to study the surface chemical states of the UiO, DAT‐HOF, and U@H2. The peak at 399.7 eV belonged to amino groups of UiO in the N 1s XPS spectra disappears in the region of the U@H2, demonstrating the conversion to the imide group.^[^ ^19^ ^]^ Significantly, the peaks assigned to Zr 3d_5/2_ and Zr 3d_3/2_ move to 182.6 and 185.0 eV (Figure 3b). This negative shift (−0.4 eV) indicates the apparent charge transfer between the UiO and DAT‐HOF in the U@H2 hybrids. As shown in Figure S12 (Supporting Information), the high‐resolution C 1s, N 1s, and O 1s XPS spectra of the U@H2 all show similar binding energy and divided peaks compared with those of the DAT‐HOF. The peak shift can also be observed in the XPS fine spectra of C1s, N1s, and O1s in the U@H2 compared with that of DAT‐HOF though the phenomenon is inconspicuous. The positive shift (+0.1 eV) corresponds well with charge transfer between the UiO and DAT‐HOF. Since the XPS technology is a surface analysis, the similar XPS spectra characteristics of the U@H2 to the DAT‐HOF corroborate the formation of the DAT‐HOF shells. The inconspicuous peak shift was probably due to the limited measurement depth of XPS analytical technique. Since the XPS technology is a surface analysis, the measurable Zr element is from the UiO coated with the HOF shell and only the small part of UiO which are closed to the HOF shell with the presence charge transfer could be measured. However, the C, N, O elements is mostly from the HOF shell, where the XPS fine spectra of C1s, N1s, and O1s mainly reflect the chemical state of the total HOF shell, thus the peak shift in U@H2 is inconspicuous compared to that of DAT‐HOF. In summary, all the characterization results suggest that the DAT‐HOF have successfully assembled on the UiO to form the U@H core–shell hybrid materials. Meanwhile, the surface functionalization step helps guide the assembly of DAT‐HOF in constructing the core–shell structures.^[^ ^10b^ ^]^ Additionally, it is worthy to note that the introduced synthesized scheme is also useful for preparing other MOF@HOF structures. For example, when NH_2_‐MIL‐68 was chosen as the host material, the final NH_2_‐MIL‐68@DAT‐HOF (MIL@H) core–shell hybrid composites were successfully yielded by the same surface functionalization and in situ self‐assembly (Scheme S1, Supporting Information). Similar results (Figures S13 and S14, Supporting Information) from analyzing the structure of MIL@H demonstrate the applicability and transferability of the synthetic method proposed here.

Antibiotic is a class of notorious organic contaminants and could cause many environmental issues.^[^ ^20^ ^]^ As the most commonly used antibiotic, TC is often detected in environmental water.^[^ ^21^ ^]^ As shown in Figure S15 (Supporting Information), the TC is of excellent chemical stability in the dark or under illumination, but it will rapidly degrade when introducing the powerful photocatalysts. In light of this, we choose it as the model pollutant to examine the photocatalytic ability of the newly formed U@H hybrid materials under visible light irradiation (*λ* > 400 nm). As shown in Figure 3c, the concentration of the TC significantly decreases within 60 min in the presence of the U@H2. Compared to pristine UiO and DAT‐HOF, the U@H2 exhibits the best photocatalytic performance with the apparent reaction rate constant of 0.0182 min^−1^, which is about 7.6 and 60.7 times larger than that of DAT‐HOF and UiO, respectively (Figure 3c). The MIL@H also shows an excellent performance in the TC degradation with the apparent reaction rate constant of 0.0175 min^−1^ relative to the poor efficiency of the MIL (Figure S16, Supporting Information). As expected, the U/H composite and the U@H3 and U@H4 show inadequately photocatalytic ability compared to the U@H2 hybrids with the apparent core–shell structure. Plus, antimicrobials (sulfonamides) and pesticides (phenylurea herbicide) were chosen in the degradation to prove the wide substrate scope in the employment of U@H2. In addition, the effects of multiple inorganic ions and organic substances were considered. The results were shown in Figure S17 in the Supporting Information.

To Figure out if the aggregation state of the HOF is responsible for the enhanced activity. The SiO_2_ was chose to be the host matrix in the construction of HOF‐based core–shell structure for its lack of photocatalytic activity. The SiO_2_@HOF hybrid was successfully constructed in the similar way and its structure was characterized (Figure S18, Supporting Information). In the photocatalytic degradation experiment, the SiO_2_@HOF hybrid even show a poorer photocatalytic activity (Figure S19, Supporting Information), which further illustrates that the aggregation state of the HOF is not contributed to the photocatalytic activity.

To explore the transfer process of electrons inside the U@H2 hybrids, electrochemical measurements were performed. As shown in the electrochemical impedance spectroscopy (EIS) Nyquist curves, the diameter of Nyquist curves in the pattern of the U@H2 is shorter compared to that of its parent materials (Figure S20, Supporting Information). In addition, the photocurrent density of U@H2 is larger than that of the UiO and DAT‐HOF. Considering thar the photocurrent measurements will create an additional interface which does not exist in the photocatalytic experiments, and the interfacial effects would somehow affect the results. To get further insight into the fundamental photophysical processes, the photoluminescence spectra and transient absorption spectroscopy (TAS) was carried out. As shown in Figure S21 (Supporting Information), when excited at 365 or 375 nm (the maximum excitation wavelength of the UiO), the emission intensity of U@H2 is weaker than that of UiO while the HOF shows no photoluminescence. As shown in Figure S22 (Supporting Information), the electronic transition type of the DAT‐HOF semiconductor is demonstrated as indirect. Usually the luminous efficiency of indirect‐type semiconductor is low, thus the DAT‐HOF in our work displays the weakest emission intensity. The weaker emission intensity of U@H2 might get benefit from the charge transfer from the UiO to the HOF.

Furthermore, the TA spectra are slightly different when HOF is introduced (Figure 3e). Compared with UiO (Figure 3d), U@H2 exhibits a weaker stimulated emission in the range shorter than 480 nm when probed at the same delay times, which implies efficient excited state quenching by HOF,^[^ ^22^ ^]^ consisting with the PL intensity and indicating that the recombination of photogeneration electron–hole pairs was suppressed.^[^ ^23^ ^]^ In addition, an absorption region from 480 to 775 nm could be found in both of the spectra while a broader region expend to the 480 nm can be found in that of UiO. The absorptive peak at around at around 650 nm could be ascribed to the photogenerated electron species of UiO and the peak at around 500 nm might be ascribed to the photogenerated electron species of HOF, suggesting it is most likely the excited state charge transfer from UiO to HOF. To further investigate the decay kinetics of the photoinduced electron‐hole pairs of these photocatalysts, the TA kinetics at 500 and 650 nm are compared (Figure S23, Supporting Information) and the fitting parameters in were shown in Table S5 in the Supporting Information. The shorter average relaxation lifetime of U@H2 than that of UiO further suggest that the efficient charge transfer from UiO to HOF.^[^ ^22^ ^]^ Plus, the TA kinetic at 500 nm indicates the extended electron lifetime in HOF result from the opening of the Z‐scheme channel, corresponding with the PL‐RL (Figure S23d, Supporting Information), which provides a higher priority and possibility for the carriers.^[^ ^24^ ^]^ Those results highlight that the hybrid core–shell structure is conducive to the effective separation of the photogenerated charges.

**Figure 4:**
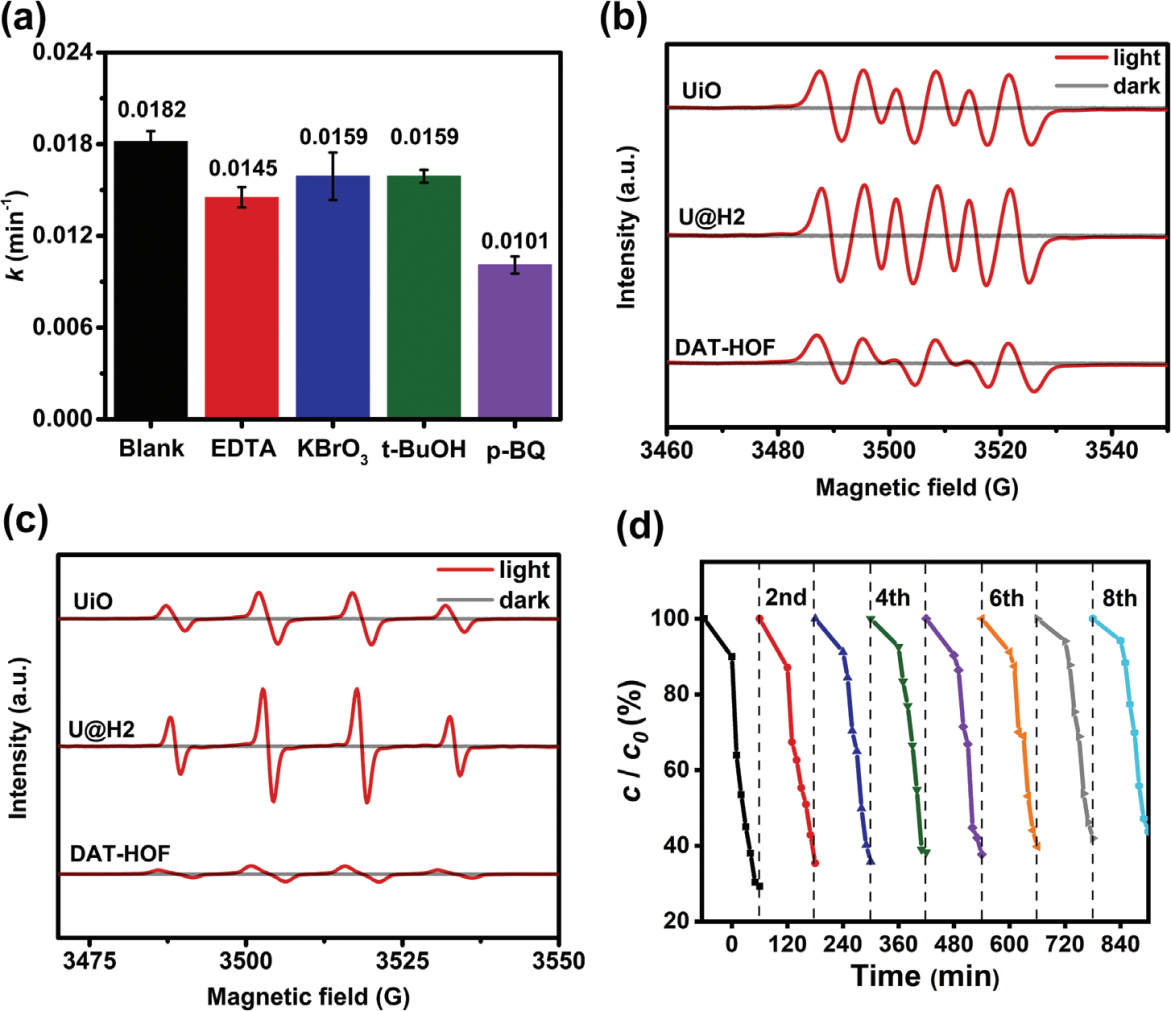
a) The apparent reaction rate constants in the presence of different scavengers (EDTA‐2Na, KBrO_3_ and TBA, p‐BQ scavenge for h^+^, e^−^, •OH, and •O_2_^−^, respectively); ESR spectra of b) DMPO–•O_2_^−^ and c) DMPO–•OH; d) Photodegradation efficiencies of TC in the presence of the U@H2 hybrid after 8 cyclic runs.

Scavenging experiments were performed to investigate the mechanism for the TC degradation using the U@H2 under irradiation. The apparent reaction rate constant decreases to 0.0101 min^−1^ in the presence of p‐benzoquinone (**Figure** **4**a), specifying that the superoxide radical (•O_2_^−^) plays a major role during the TC degradation. The dominance of •O_2_^−^ is further supported by the varied TC degradation rates when an electron (e^−^) was quenched. Additionally, the apparent reaction rate constants could slightly decrease in the presence of ethylenediaminetetraacetic acid disodium salt (EDTA) and tert‐butanol, implying the secondary importance of the hole (h^+^) and hydroxyl radical (•OH). To probe the intrinsic reactive mechanism, electron paramagnetic resonance (EPR) spin‐trapping technology was executed with 5,5‐dimethyl‐1‐pyrroline *N*‐oxide (DMPO) as a spin trap (Figure 4b,c). Compared with the DAT‐HOF and pristine UiO, the stronger intensity of characteristic peaks related to DMPO–•O_2_^−^ and DMPO–•OH in the EPR spectra suggests that the DAT‐HOF shell could accelerate the generation of more reactive oxygen species under irradiations. In light of this, the separation efficiency of charge carriers of the U@H2 is enhanced by producing more reactive oxygen species due to the core–shell heterojunction. Furthermore, the core–shell hybrid materials inherit the chemical stability of their parent materials. The soaked U@H2 in different solvent still show unchanged crystallinity and structure (Figure S24, Supporting Information). And there was no dissolution observed in many common solvents (Figure S25, Supporting Information). The photocatalytic performance of the newly derived U@H2 hybrid is stable and effective even after eight cyclic runs of the photostability tests (Figure 4d). The stable structure of the used U@H2 has been well illustrated by the Figures of TEM, PXRD, FTIR, and BET (Figure S26a–e, Supporting Information).

**Figure 5:**
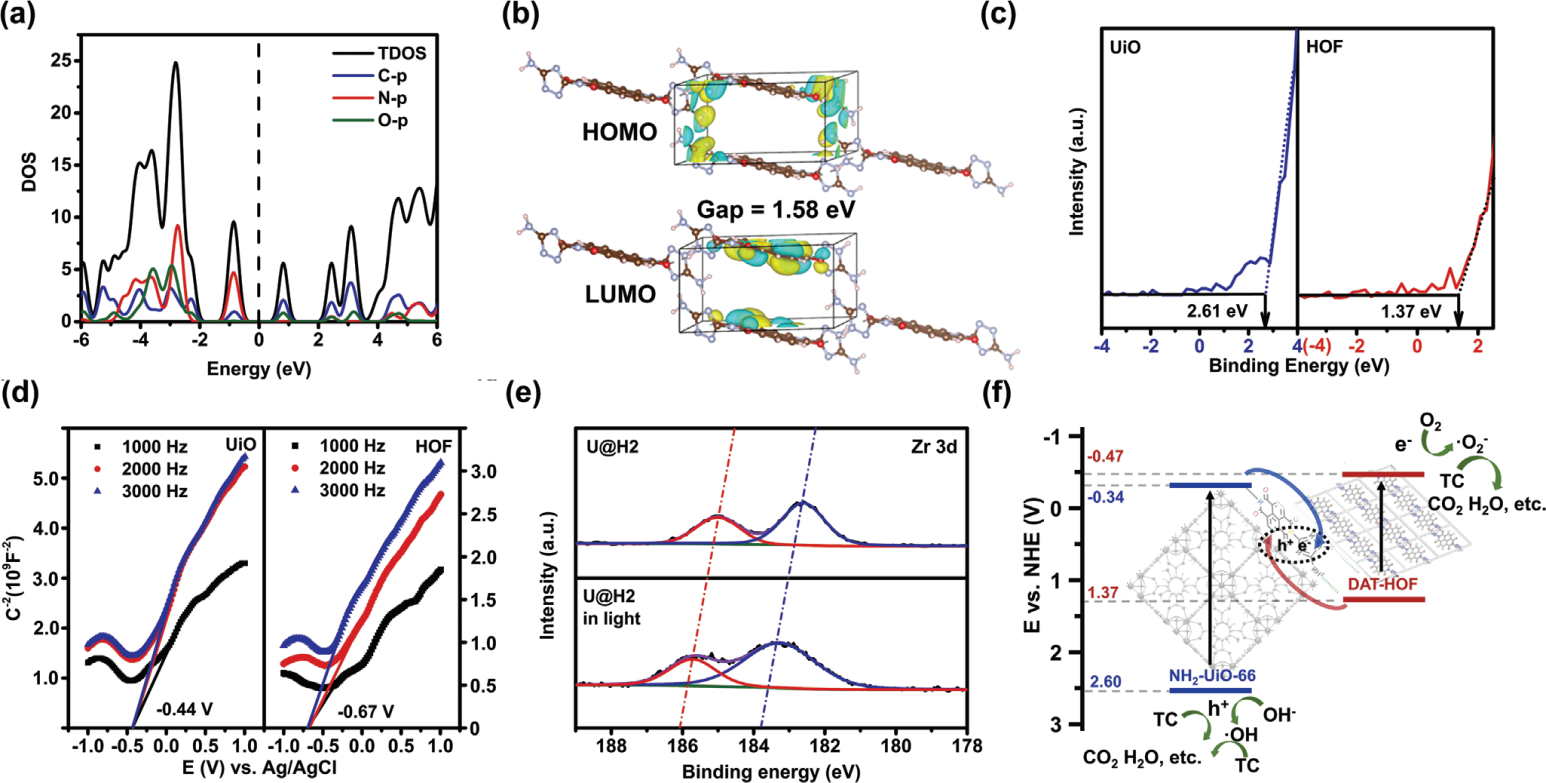
a) Density of states (DOS) and b) HOMO and LUMO levels of the HOF; c,d) XPS valence band spectrum and Mott–Schottky plots of the UiO and HOF; e) In situ XPS spectra of the U@H2; f) Energy band structure of the U@H2 heterojunction.

The in‐depth investigation of the inner electron transfer mechanism and the confirmation of the heterojunction were implemented using the band structure‐related parameters obtained through the theoretical calculation and a series of characteristic experiments. The corresponding theoretical bandgap is 1.58 eV, close to the bandgap (1.84 eV) calculated according to the Tauc plot (Figure 3a). Furthermore, the density of states (DOS) analysis reveals that the highest occupied molecular orbital (HOMO) level of the DAT‐HOF is constituted by the N and C atoms, while the lowest unoccupied molecular orbital (LUMO) level is composed of the O and C atoms (**Figure** **5**a). They correspond well with the schematic diagram of the HOMO and LUMO orbitals illustrated in Figure 5b, where the HOMO/LUMO orbitals are mainly distributed on the DAT/NTCDA molecules. The spatial separation of HOMO and LUMO benefits from restraining the recombination of charge carriers, thereby foreshadowing its great potential in photocatalysis. The flat band potentials for the UiO and DAT‐HOF are −0.24 and −0.37 V (vs NHE), respectively. Considering that the flat band potential is normally positive (0.1–0.2 V) than the conduction band (CB) bottom potential,^[^ ^25^ ^]^ the CB potentials for the UiO and DAT‐HOF can be approximated as −0.34 and −0.57 V (vs NHE, Figure 5d). Based on the bandgap (2.94 and 1.84 eV for the UiO and DAT‐HOF, respectively) estimated by the Tauc plot, the valence band (VB) potential can be calculated as 2.60 and 1.27 V (vs NHE) for the UiO and DAT‐HOF. In addition, we also consider directly using the VB‐XPS method following formula:^[^ ^26^ ^]^ *E*_VB_, _NHE_ =  *φ* + *E*_VB, XPS_ − 4.44, Thus, the *E*_VB_ of UiO and HOF were calculated to be 2.67 and 1.43 eV (The work function of the instrument *φ* = 4.5 eV). The values calculated by those two methods are close to each other. Considering that the direct VB‐XPS method could get more accurate band position, we use the value calculated by the direct VB‐XPS method, i.e., 2.67 eV for UiO and 1.43 eV for HOF, as the valence band potential of the individual components of U@H. Accordingly, the energy band structures of the U@H2 hybrids are illustrated in Figure 5f. Thus, under irradiation, the electrons photogenerated from the photoactive ligands of the UiO prefer to transfer to the DAT‐HOF shell due to the lower VB potential, matching the S‐scheme charge transfer mechanism. The positive shift of binding energy for Zr 3d in the in situ XPS analysis can further confirm this (Figure 5e).^[^ ^27^ ^]^ The S‐scheme heterojunction can facilitate the separation of charge carriers and contribute to the production of active species.

To further Figure out the active sites and confirm the charge transfer route, the metal photo deposition experiments were performed. As shown in Figure S27 (Supporting Information), the successful deposition of Au particles instead of PbO_2_ on U@H2 surface indicated that the photogenerated electrons were left on the U@H2 surface while the holes were not. This further illustrate that the electrons remain at HOF shell and holes are might located at the UiO core in U@H2 hybrid, corresponding well with the charge transfer route in Figure 5f.

## 3 Conclusion

In this study, we proposed a universal and effective synthesis scheme that can be applied to generate the MOF@HOF core–shell hybrid materials exhibiting added photocatalytic efficiency compared to either individual MOF or HOF. Two core steps in constructing this unique core–shell nanostructure include the aforehand functionalization of MOF core and the subsequently reasonable interfacial growth of HOF shell through being soaked into the DAT and NTCDA solutions of proper concentrations. Either the absence or misconduct of the above procedures likely led to the formation of MOF@HOF composites without the core–shell structures and/or considerable reduction in the photocatalytic capability of the hybrids.

Here, we designed and synthesized DAT‐HOF‐based core–shell materials (NH_2_‐UiO‐66@DAT‐HOF) using a batched of concentrations of the DAT‐HOF. These newly yielded U@H composites could display both characteristic peaks of the UiO and DAT‐HOF in the FT‐IR and XRD spectrum, and their particular core–shell structures can be clearly observed in the TEM and SEM images. Experimental results have demonstrated a significant enhancement in terms of the photocatalytic ability of these hybrid materials compared to their parent particles. Specifically, the apparent reaction rate of the U@H2 in tetracycline degradation could reach 0.0182 min^−1^, which is 60.7 and 7.6 times higher than that of the parental UiO and DAT‐HOF. Such a significant improvement in the photocatalytic efficiency could be majorly attributed to the peculiar structure of the DAT‐HOF shell coated on the MOFs core, which not only extends the utilization range of the visible light but also improves the hole‐electron pair's separation because of the S‐scheme heterojunction. Furthermore, the transferability of the proposed synthesis framework has been confirmed by producing other MOF@HOF core–shell materials, i.e., NH_2_‐MIL‐68@DAT‐HOF. From a broad perspective, in addition to the provision of a novel strategy to effectively integrate the MOFs and HOFs, the outstanding performance of the advanced U@H materials in the TC photocatalytic degradation offers a practically viable tool for the removal of contaminants, making a significant potential to water quality improvement as well as ecosystem sustainability.

## Conflict of Interest

The authors declare no conflict of interest.

## Supporting information

Supporting Information

## Data Availability

The data that support the findings of this study are available from the corresponding author upon reasonable request.
