# Supplementary material for: In Situ Assembly of Hydrogen‐Bonded Organic Framework on Metal–Organic Framework: An Effective Strategy for Constructing Core–Shell Hybrid Photocatalyst
Source: Adv Sci (Weinh). 2022 Oct 18;9(34):2204036. doi: 10.1002/advs.202204036 (PMC9731681; doi:10.1002/advs.202204036)
Supplement: Supplementary file 1 — Supporting Information [file ADVS-9-2204036-s001.pdf]

Supporting Information

In Situ Assembly of Hydrogen-bonded Organic Framework on Metal-organic Framework: An Effective Strategy for Constructing Core–Shell Hybrid Photocatalyst

Jianli Wang[1], Yifan Mao[2], Runze Zhang[3], Yanli Zeng[4], Changsheng Li[5], Bingjie Zhang[1], Jianhui Zhu[1], Jiawen Ji[1], Desheng Liu[1], Rumin Gao[1], Yongqiang Ma[1]*

[1] Department of Applied Chemistry, College of Science, China Agricultural University, Beijing, 100193, P.R. China

[2] Department of Chemistry, University of Virginia, Charlottesville, Virginia, VA 22094, USA

[3] Department of Engineering Systems and Environment, University of Virginia, Charlottesville, Virginia, VA 22904, USA

[4] College of Chemistry and Materials Science, Hebei Normal University, Shijiazhuang, 050024, P.R. China

[5] College of Agronomy and Biotechnology, China Agricultural University, Beijing, 100193, P.R. China

Tel number: +86-010-62731978

E-mail: mayongqiang@cau.edu.cn

1. Experimental Section

***Chemicals*:** Acetic acid (HAc, 99.8%), tert-Butanol (TBA, 98%) Potassium bromate ($KBrO_3$, 99.8%), Ethylenediamine tetraacetic acid disodium salt (EDTA-2Na, 99%), methanol (99.7%) and ethanol (99.5%) were purchased from Sinopharm Chemical Reagent Co., Ltd., China. 1,4,5,8-Naphthalenetetracarboxylic dianhydride (NTCDA, 97.5%), 2-aminoterephthalic acid (H_2ATA, 98%), Indium(III) nitrate hydrate ($In(NO_3)_3 \cdot \chi H_2O$, 99.99%), N, N-Dimethylacetamide (DMAc, 99%), N, N-Dimethylformamide (DMF, 99.9%), Tetracycline hydrochloride (TC, 98%), p-Benzoquinone (p-BQ, 99%), 3,5-Diamino-1,2,4-triazole (DAT, 98%), chlorotoluron (99%), isoproturon (99%), sulfadiazine (99%), sulfathiazole (98%), sulfapyridine (99%) and sulfamethazine (99%) were purchased from J&K Scientific Ltd., China. Zirconium (IV) chloride ($ZrCl_4$, 99.5%) was purchased from Strem Chemicals, Inc. sulfamethoxydiazine (98%) was purchased from Macklin Inc., China. sulfamerazine (98%) was purchased from Alfa Aesar Chemical Co., Ltd., China. metoxuron (99%), diuron (99%) was purchased from Shanghai Aladdin Biochemical Technology Co., Ltd. Deionized water was used in the photocatalytic degradation of TC.

***Preparation of NH_2-UiO-66 (UiO)*:** $ZrCl_4$ (61.2 mg, 0.26 mmol) was dissolved in 60 mL of DMF in a conical bottle with stopper, then H_2ATA (87.0 mg, 0.48 mmol) was added and the solution was sonicated until the solution becomes clear. 7.2 mL of HAc was added and the solution was stirred, then the mixture was transferred into a 100 mL Teflon-lined stainless-steel autoclave and heated at 120 °C for 12 hours. After cooling to room temperature, the resulted solid was washed with DMF and ethanol, then lyophilized for 24 hours.

***Preparation of DAT-HOF (HOF)*:** DAT (99.1 mg, 1.00 mmol) was dissolved in 10 mL of DMAc in an ice bath under N_2 for 30 min, then NTCDA (134 mg, 0.50 mmol) was added and the mixture was stirred vigorously in an ice bath for another 30 min. Subsequently, the mixture was diluted with 60 mL of DMAc, followed by stirring in an ice bath for 5 min. The above solution was transferred into a 100 mL Teflon-lined

stainless-steel autoclave and heated at 170 °C for 6 hours. After cooling to room temperature, the resulted solid was washed with DMF and methanol, then lyophilized for 24 hours.

***Preparation of NH_2-UiO-66@DAT-HOF core-shell hybrid material (U@H)*:** In a typical reaction, NTCDA (268.2 mg, 1.00 mmol) and the obtained UiO (200 mg) were dissolved in 10 mL of HAc. Then, the mixture was stirred vigorously and heated at 119 °C for 16 hours. Upon cooling, the resulted solid was washed with DMF and methanol, then lyophilized for 24 hours to obtain anhydride functionalized NTCDI-UiO (N-UiO). In step two, DAT (99.1 mg, 1.00 mmol) was dissolved in 10 mL of DMAc in an ice bath under N_2 for 30 min, then NTCDA (134.1 mg, 0.50 mmol) was added and the mixture was stirred vigorously in an ice bath for another 30 min. Subsequently, the obtained N-UiO and 60 mL of DMAc were added into the mixture, followed by stirring in an ice bath for 5 min. The above was transferred into a 100 mL Teflon-lined stainless-steel autoclave and heated at 170 °C for 6 hours. After cooling to room temperature, the resulted solid was washed with DMF and methanol, then lyophilized for 24 hours to obtain U@H2.

The preparation of U@H1 was similar to that of U@H2 but using 49.5 mg DAT and 67 mg NTCDA.

The preparation of U@H1.5 was similar to that of U@H2 but using 66.8 mg DAT and 100.5 mg NTCDA.

The preparation of U@H2.5 was similar to that of U@H2 but using 111.3 mg DAT and 167.5 mg NTCDA.

The preparation of U@H3 was similar to that of U@H2 but using 198.2 mg DAT and 268.2 mg NTCDA.

The preparation of U@H4 was similar to that of U@H2 but using 297.3 mg DAT and 402.3 mg NTCDA.

***Preparation of NH_2-UiO-66/DAT-HOF material (U/H)*:** The preparation of U/H was similar to that of U@H2 but using pristine UiO instead of N-UiO.

***Preparation of NH_2-MIL-68 (MIL)*:** H_2ATA (45.3 mg, 0.25 mmol) and BA (7.3 mg, 0.06 mmol) were dissolved in 1.5 mL of DMF in a 10 mL capped vial then 40 μL of pyridine was added. $In(NO_3)_3{\cdot}\chi H_2O$ (105.0 mg, 0.35 mmol) dissolved in 1 mL of DMF was added. The mixture was sonicated for 5 min and heated at 125 °C for 2.5 h. Upon cooling, the yellow powder was washed with DMF and ethanol, then lyophilized for 24 hours.

***Preparation of NH_2-MIL-68 @DAT-HOF core-shell hybrid material (MIL@H)*:** In a typical reaction, NTCDA (268.2 mg, 1.00 mmol) and the obtained MIL (200 mg) were dissolved in 10 mL of HAc. Then, the mixture was stirred vigorously and heated at 119 °C for 5 hours. Upon cooling, the resulted solid was washed with DMF and methanol, then lyophilized for 24 hours to obtain anhydride functionalized MIL. In step two, DAT (99.1 mg, 1.00 mmol) was dissolved in 10 mL of DMAc in an ice bath under N_2 for 30 min, then NTCDA (134.1 mg, 0.50 mmol) was added and the mixture was stirred vigorously in an ice bath for another 30 min. Subsequently, the obtained anhydride functionalized MIL and 60 mL of DMAc were added into the mixture, followed by stirring in an ice bath for 5 min. The above was transferred into a 100 mL Teflon-lined stainless-steel autoclave and heated at 170 °C for 6 hours. After cooling to room temperature, the resulted solid was washed with DMF and methanol, then lyophilized for 24 hours to obtain MIL@H.

All the yields of samples were given and presented in Table S1.

***Photocatalytic experiments*:** The photocatalytic degradation experiments were carried out under Xe lamp irradiation with a cut-off filter (300 W, Light intensity = 100 mW cm^{-2}, $\lambda > 400$ nm). 20 mg photocatalyst was added to 100 mL of TC solution (50 mg L^{-1}) and the suspension was stirred in the dark for 60 min before irradiation. Then the Xe lamp was turned on and 1.0 mL of the suspension was taken out per 10 minutes. EDTA-2Na (10 mM), TBA (10 mM), $KBrO_3$ (10 mM) and p-BQ (0.5 mM) were used as scavengers for holes, hydroxyl radicals, electrons and superoxide radicals in the

scavenging experiments, respectively.

Metal photodeposition experiments: Photodeposition of Au and PbO_2 on the surfaces of U@H2 were carried out using H_2AuCl_4 and $Pb(NO_3)_2$ as precursors, respectively. Typically, 20 mg of U@H2 and the metal precursor were mixed in 100 mL of solvent with stirring. In the photo deposition of Au, 2 mg of H_2AuCl_4 was added and the mixture of distilled water (80 mL) and isopropanol (20 mL), then the precursor was degassed and bubbled with N_2. The suspension was then irradiated by a 300 W Xe lamp. After 4 h of photo deposition, the suspension was filtered, washed several times with deionized water, and finally dried in the oven at 60 °C overnight. In the photo deposition of PbO2, 2 g of $NaIO_3$ and 1 mL of $Pb(NO_3)_2$ (40 mg L^{-1}) was added to distilled water (100 mL). The suspension was then irradiated by a 300 W Xe lamp. After 4 h of photo deposition, the suspension was filtered, washed several times with deionized water, and finally dried in the oven at 60 °C overnight.

Preparation of SiO_2@HOF core-shell hybrid material: The amino-functionalized SiO_2 (NH_2-SiO_2) was synthesized according to the literature.[1] In a typical reaction, NTCDA (268.2 mg, 1.00 mmol) and the obtained NH_2-SiO_2 (200 mg) were dissolved in 10 mL of HAc. Then, the mixture was stirred vigorously and heated at 119 °C for 5 hours. Upon cooling, the resulted solid was washed with DMF and methanol, then lyophilized for 24 hours to obtain anhydride functionalized SiO_2. In step two, DAT (99.1 mg, 1.00 mmol) was dissolved in 10 mL of DMAc in an ice bath under N_2 for 30 min, then NTCDA (134.1 mg, 0.50 mmol) was added and the mixture was stirred vigorously in an ice bath for another 30 min. Subsequently, the obtained anhydride functionalized SiO_2 and 60 mL of DMAc were added into the mixture, followed by stirring in an ice bath for 5 min. The above was transferred into a 100 mL Teflon-lined stainless-steel autoclave and heated at 170 °C for 6 hours. After cooling to room temperature, the resulted solid was washed with DMF and methanol, then lyophilized for 24 hours to obtain SiO_2@HOF.

Analytical methods**:** The samples were filtered through a microfiltration membrane (13 mm*0.22 μm) before analysis on the high-performance liquid chromatography (HPLC, Agilent 1100). The HPLC equipped with a UV-vis detector at a wavelength of 347 nm and Agilent Extend SB-C18 column (5 μm particle size, 4.6 mm × 250 mm). The mobile phase was consisted of H_2O (containing 0.1% formic acid) and acetonitrile at a flow rate of 1 mL min^{-1}, and the ratio of H_2O and acetonitrile was 88:12.

A pseudo-first-order kinetic model was used and calculated by Eq. (1) to further compare the photocatalytic efficiencies.

$$\ln(C_0/C_t) = k\mathrm{t} \qquad \text{Eq. (1)}$$

where C_0 and C_t are the initial concentration and the remaining concentration, respectively, of TC (mg L^{-1}) at each time point; k is the kinetics rate constant; and t is the reaction time (min). the fitting results of the pseudo-first-order kinetic model in each degradation were supplied in Table S2.

Characterization**:** The crystal phase identification of photocatalysts were conducted via an X-ray diffractometer (XRD, Bruker D8 Advanced diffractometer) using Cu-Kα radiation (λ = 1.5406 Å) in the 2θ range from 2° to 55°. The microstructures and morphologies were analyzed by microscopy (Hitachi UHR FE-SEM SU8020 scanning electron microscopy and JEM-1200EX transmission electron microscopy). The molecular structure was analyzed by Fourier transform infrared spectroscopy (FT-IR, Thermo Scientific Nicolet iS10 FT-IR Spectrometer). The surface areas were carried out by Micromeritics ASAP 2020 using the Brunauer-Emmett-Teller (BET) equation. Meanwhile, the chemical state was analyzed by X-ray photoelectron spectrometer (Thermo Scientific Escalab 250Xi X-ray photoelectron spectrometer). UV-visible-near infrared light spectrophotometer (Shimadzu UV-3600 Spectrophotometer) was used to get the light absorption of photocatalysts. The photoluminescence of samples was detected on a spectrofluorometer (PL, FLS1000/FS5 Spectrofluorometer) with an excitation wavelength of 380 nm. The

thermogravimetric analysis (TGA) tests were performed on the thermogravimetric analyzer (NETZSCH STA 449 F5/F3 Jupiter). The ESR signals of samples were recorded on a spectrograph (Bruker A300) using 5,5-Dimethyl-1-pyrroline-N-oxide (DMPO) as the spin trap. The C, N, O elemental contents were detected on elemental analyzer (Elementar Vario EL cube), The Zr elemental content was detected on elemental analyzer (ICP-MS, Agilent ICPMS7800).

All the electrochemical measurements were performed in a conventional three-electrode cell on a CHI-760E electrochemical workstation (Shanghai Chenhua Instrument Co., Ltd, China). During the photocurrent measurement, an Ag/AgCl electrode was used as the reference electrode and a Pt foil electrode acted as the counter electrode. The working electrodes were designed using resulting samples covered on the surface of fluoride tin oxide (FTO) conductor glass. A quartz cell filled with 0.5 M Na_2SO_4 (pH = 6.8) electrolyte was used as the measure system. For electrochemical impedance spectroscopy (EIS) measurements, the amplitude of the sinusoidal wave was 5 mV, and the frequency range from 100 kHz to 0.05 Hz.

Theoretical Calculation**:** All calculations were carried out by using the Vienna ab initio simulation package (VASP), where the generalized gradient approximation (GGA) and the projected augmented wave potentials (PAW) with 500 eV cutoff energy have been employed. The exchange and correlation function has been approximated to be the Perdew-Burke-Ernzerhof parametrization (PBE). For the structural optimizations, we used the Monkhorst-Pack k-point meshes of 3*3*5 and the converging criterions of 0.01 eV/Å for the force and 10-5 eV for the energy, respectively. Band structure and projected density of states (PDOS) was calculated by hybrid functional calculation using the HSE06 functional.

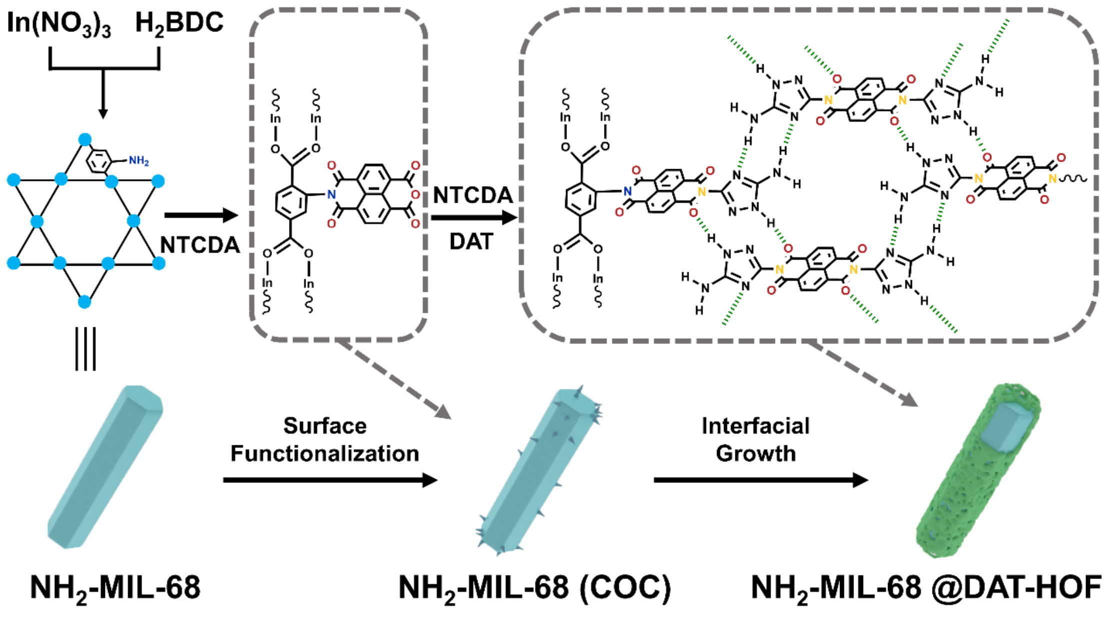

Scheme S1. Illustration of the synthetic strategies for NH_2-MIL-68@DAT-HOF photocatalysts.

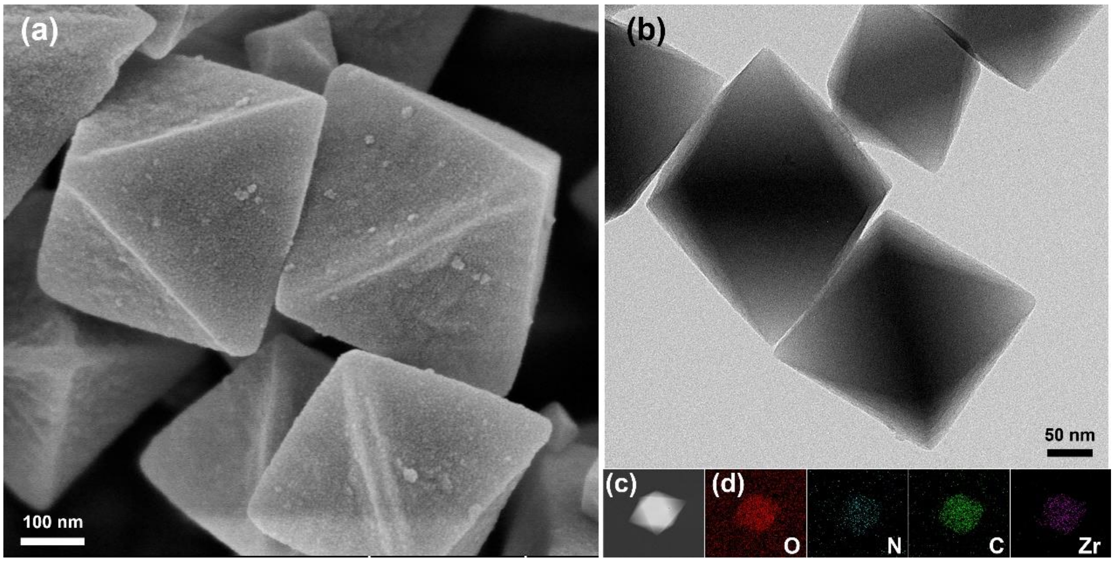

Figure S1. (a) SEM image; (b) TEM image; (c) HAADF-STEM image and (d) Elemental mapping images of pristine UiO.

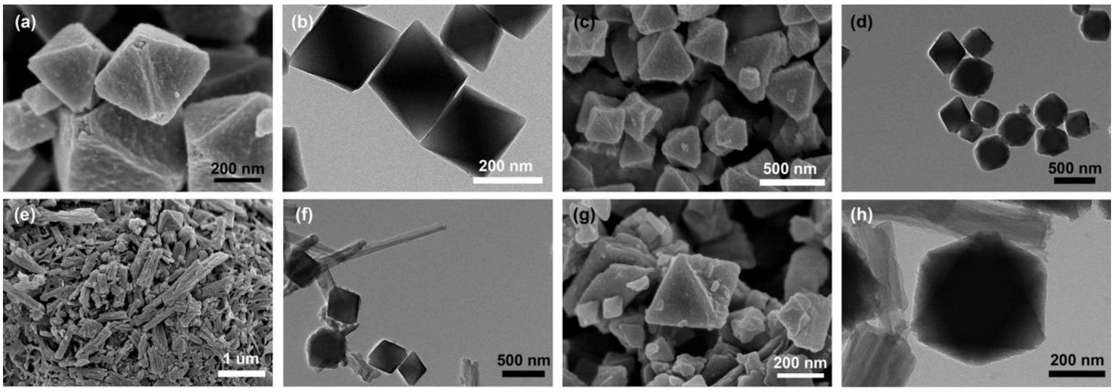

Figure S2. (a) SEM and (b) TEM images of NTCDI-UiO (N-UiO); (c) SEM and (d) TEM images of Blank-UiO; (e, g) SEM and (f, h) TEM images of U/H.

Note: The Blank-UiO was prepared in the same solvothermal conditions (170°C for 6 hours) without any NTCDA and DAT added. As can be seen in Figure S2b, after the solvothermal process, the main framework of UiO is retained with slight dent and part of which surround ed by small fragments.

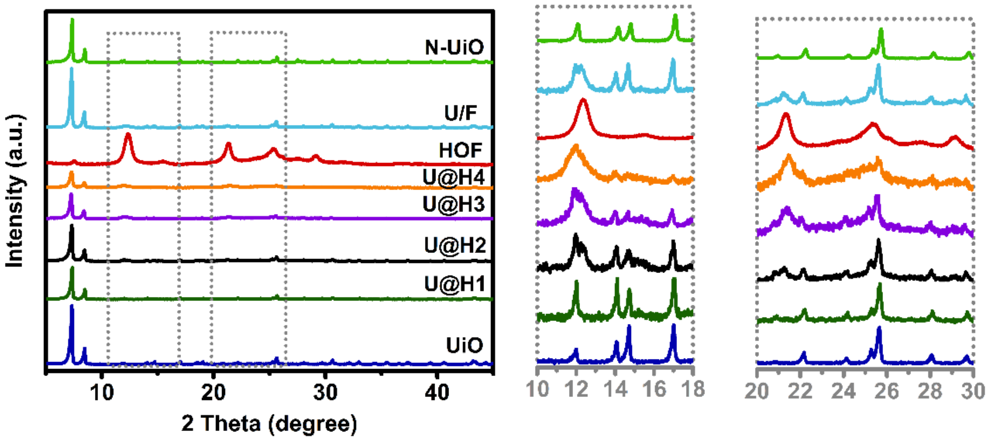

Figure S3. XRD pattern of the as-synthesized samples.

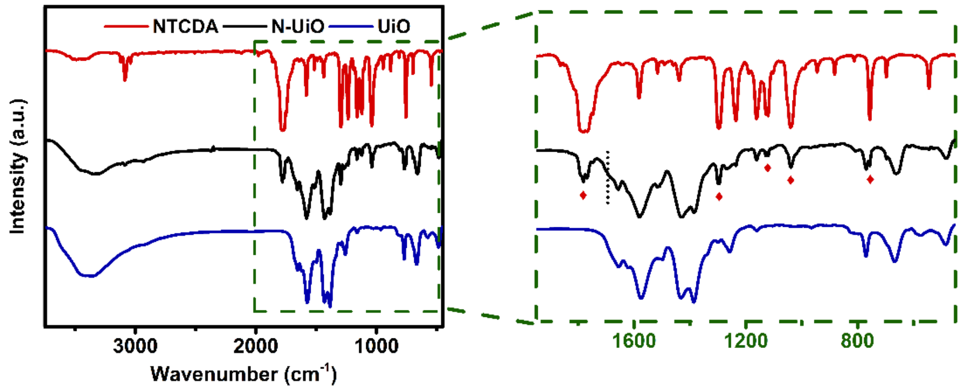

Figure S4. FT-IR spectrum of NTCDA, N-UiO and UiO.

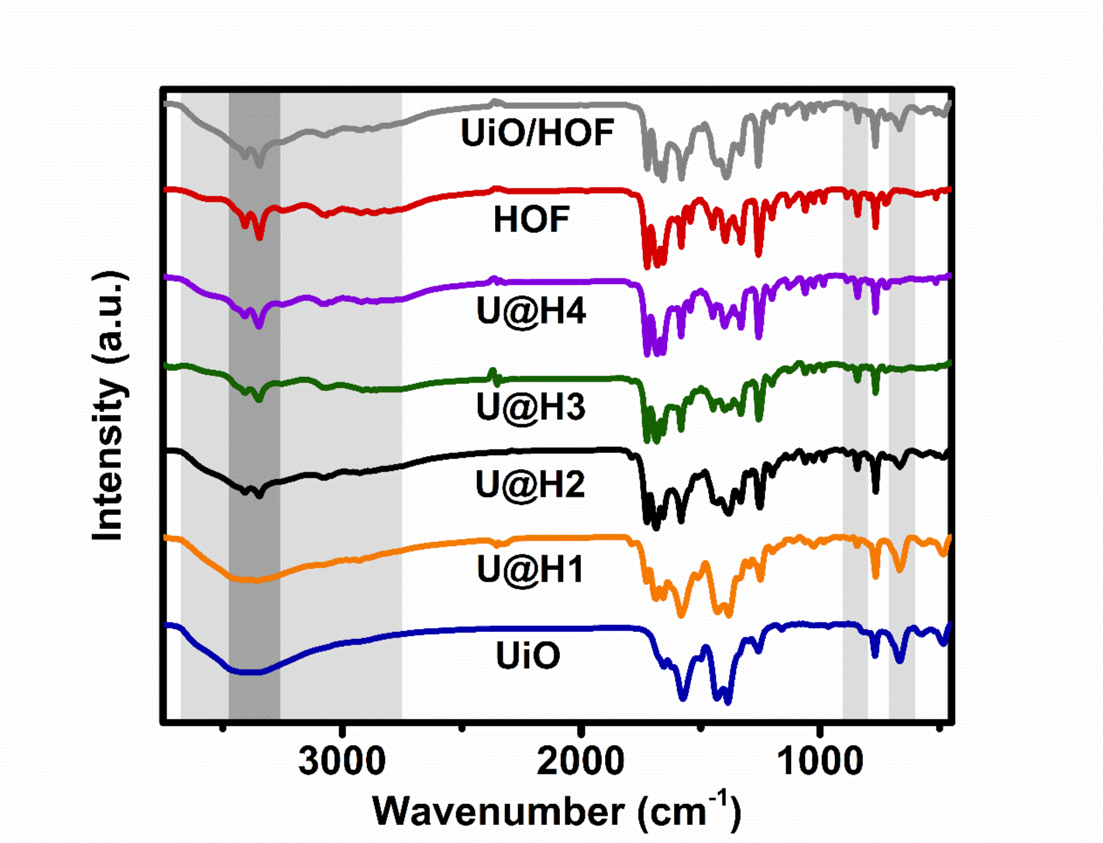

Figure S5. FT-IR spectrum of the as-synthesized samples.

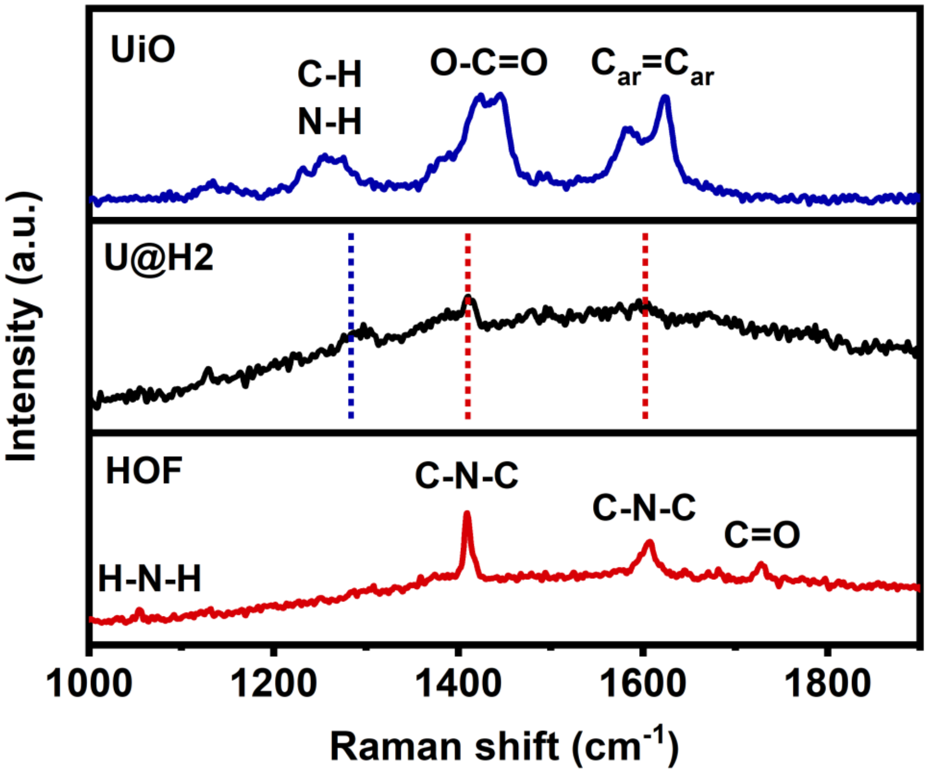

Figure S6. Raman spectra of UiO, U@H2 and HOF.

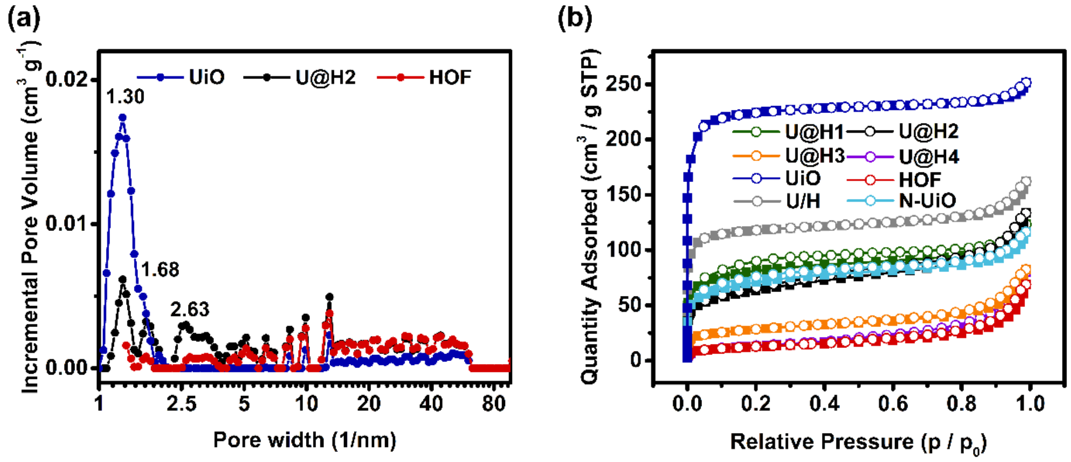

Figure S7. (a) Pore size distributions of UiO, U@H2 and HOF; (b) N_2 adsorption-desorption curves of the as-synthesized samples.

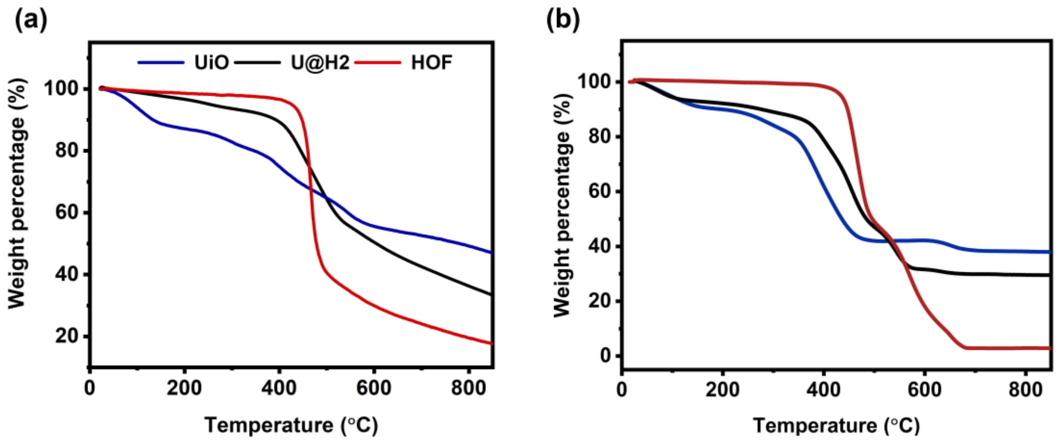

Figure S8. TGA curves under N_2 (a) and O_2 (b).

Note: HOF was complete combusted at around 700 °C. After heating at 800°C, the residual amount for the MOF is 36.8% (ZrO_2), and the residual amount for the U@H2 is 29.5% (ZrO_2). The content of HOF in U@H2 is calculated to be 19.8% by the thermogravimetric analysis (1-29.5%/36.8%).

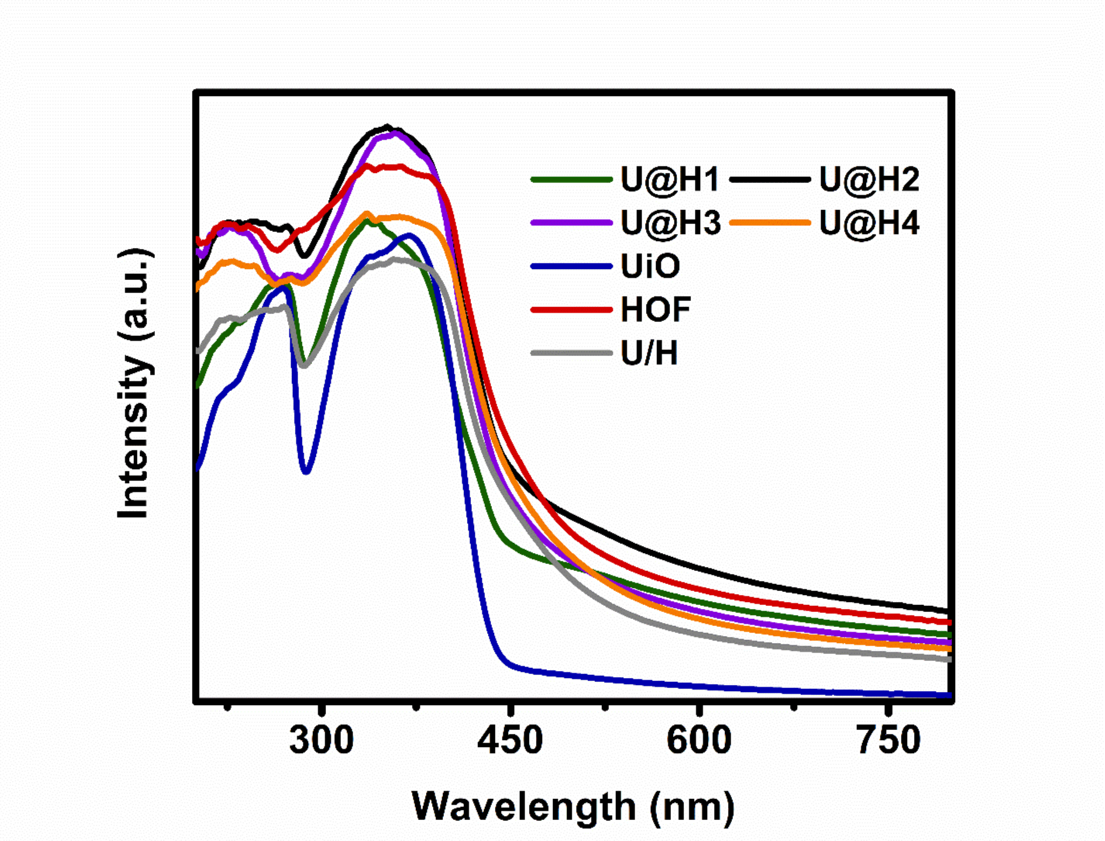

Figure S9. UV-Vis DRS spectra of the as-synthesized samples.

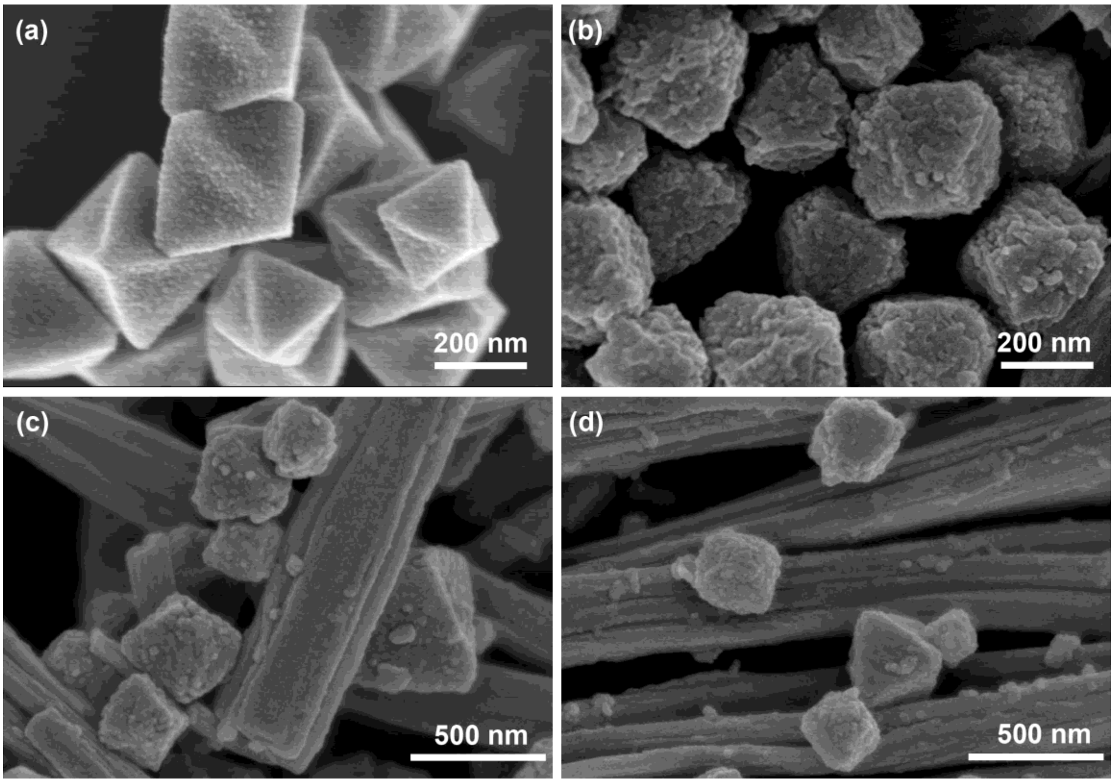

Figure S10. (a-d) SEM images of U@H1, U@H2, U@H3 and U@H4.

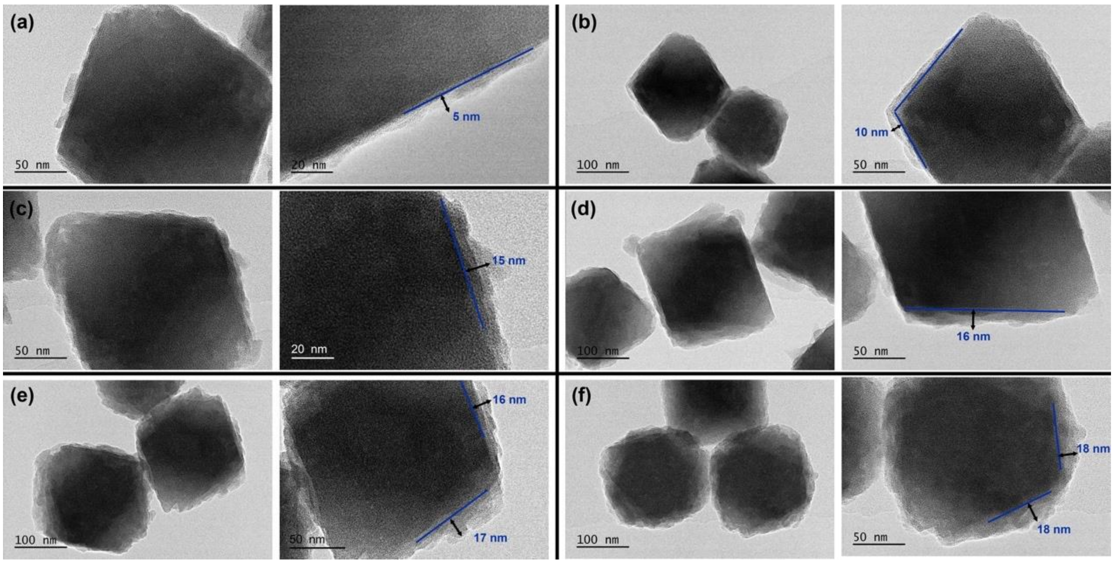

Figure S11. TEM images of the as-synthesized samples: (a) U@H1, (b) U@H1.5, (c) U@H2, (d) U@H2.5, (e) U@H3 and (f) U@H4.

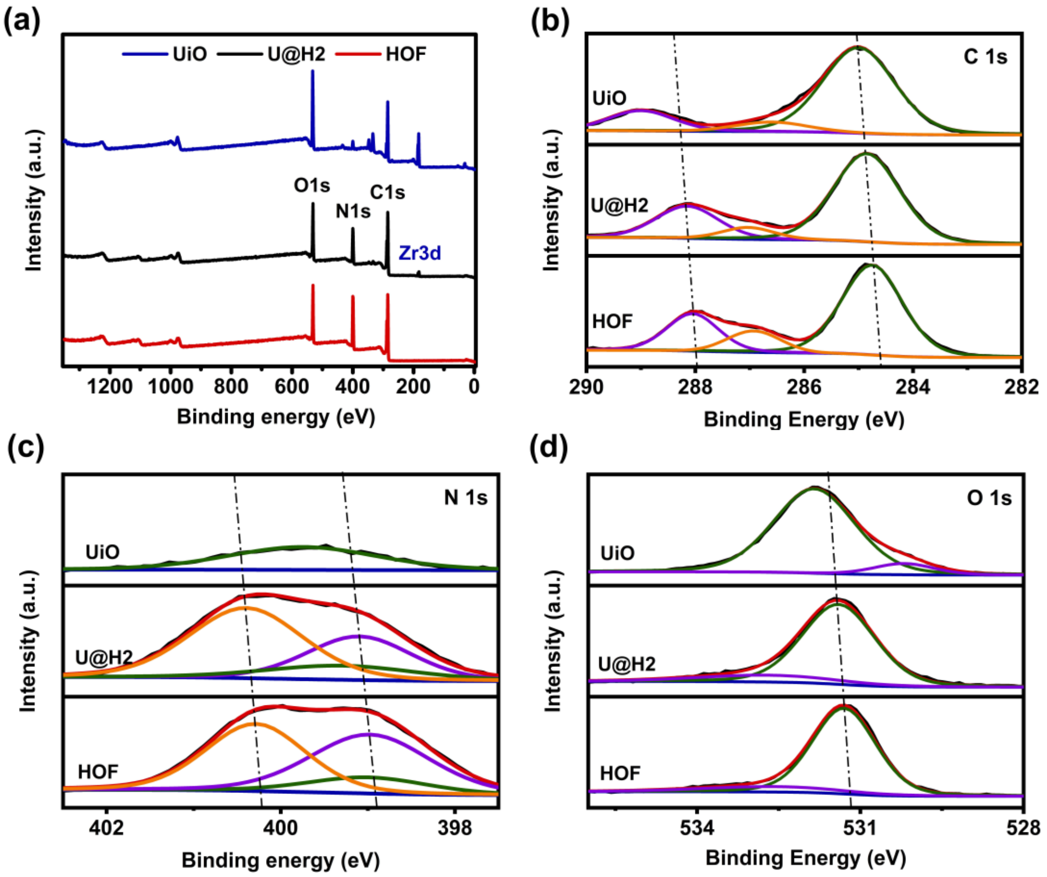

Figure S12. (a) XPS spectra; High-resolution (b) C 1s, (c) N 1s and (d) O 1s XPS spectrum of UiO, U@H2 and HOF.

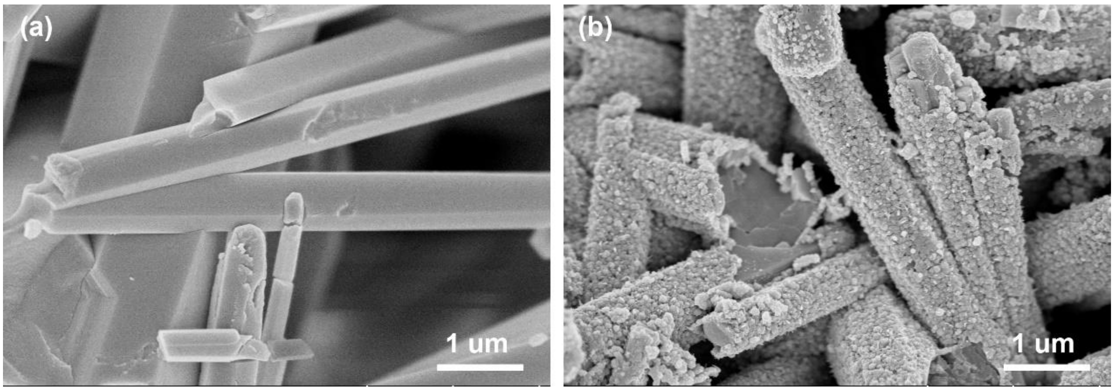

Figure S13. SEM images of (a) pristine MIL and (b) MIL@H.

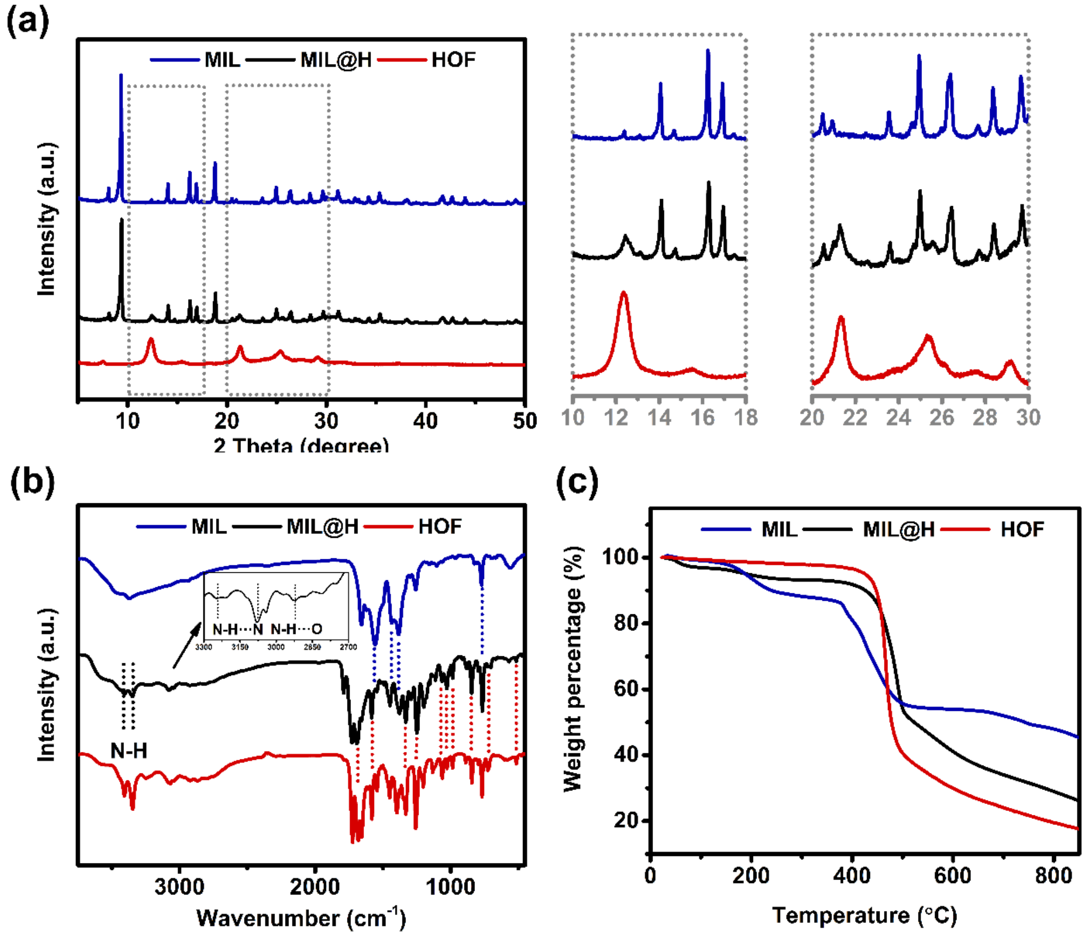

Figure S14. (a) XRD pattern, (b) FT-IR spectrum and (c) TGA curves of the MIL, HOF and MIL@H.

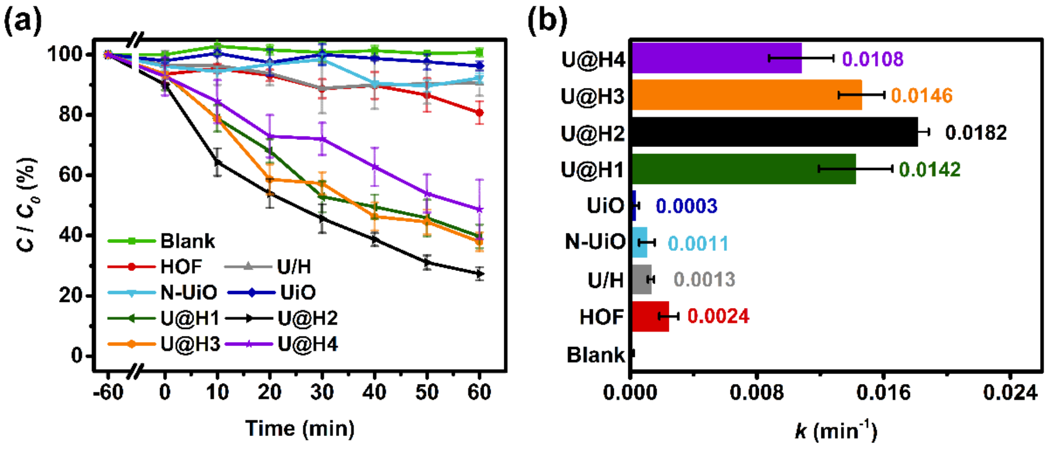

Figure S15. (a) Comparisons of photodegradation efficiencies of TC in the presence of different photocatalysts; (b) The apparent reaction rate constants for different photocatalysts.

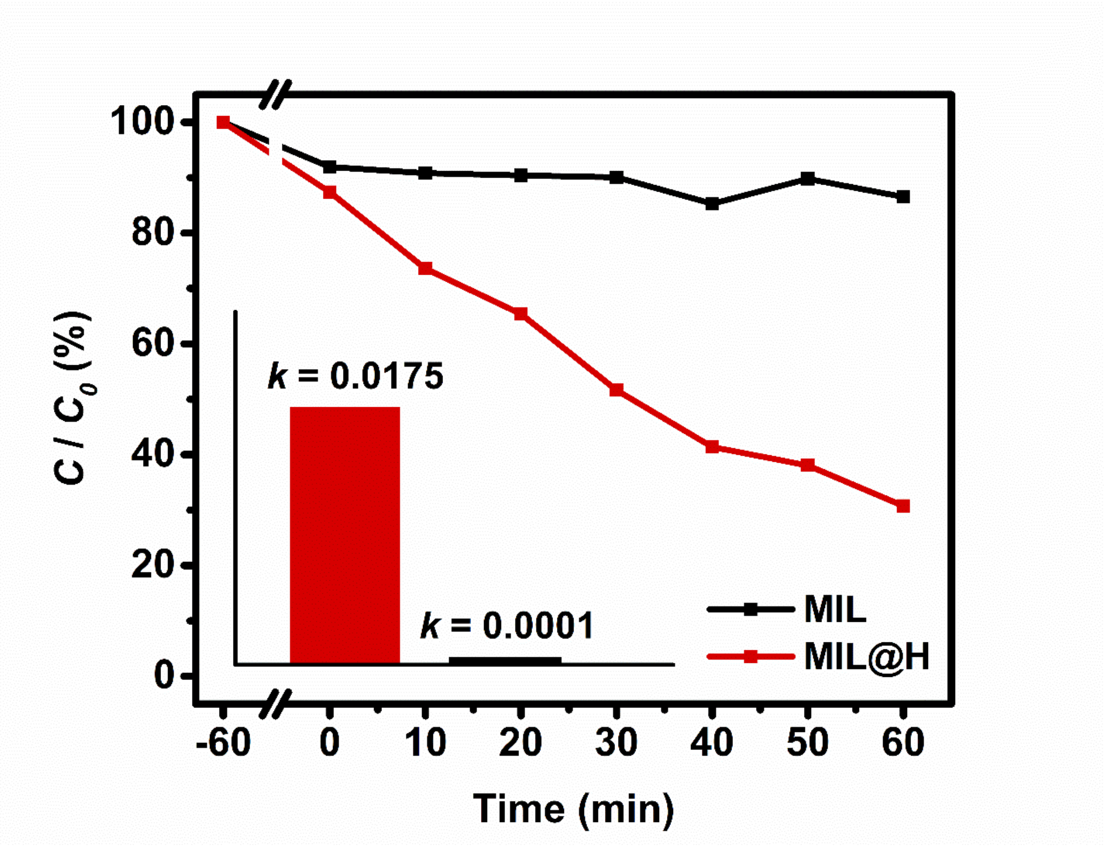

Figure S16. Comparisons of photodegradation efficiencies of TC and the apparent reaction rate constants (inset) in the presence of MIL and MIL@H.

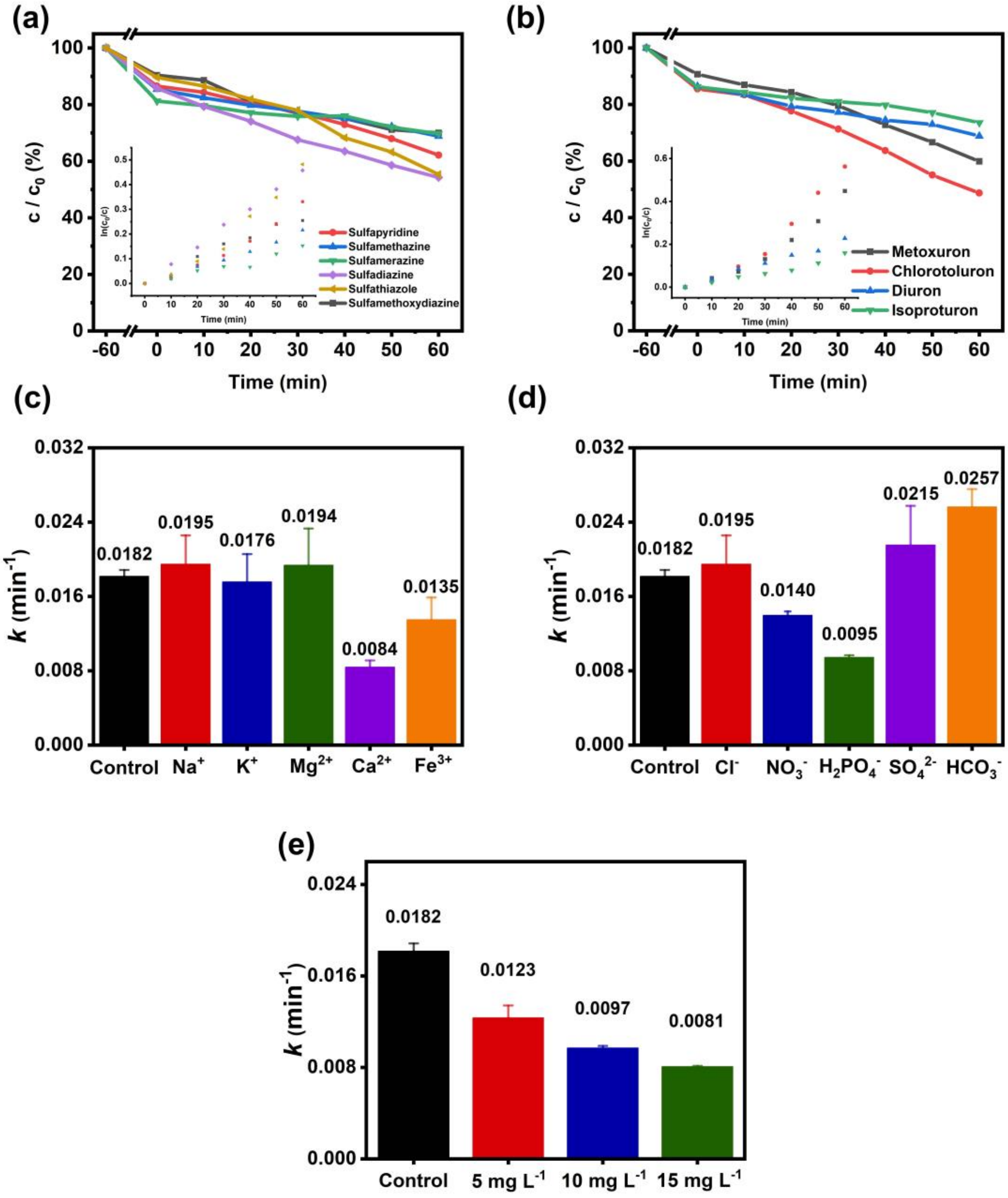

Figure S17. The degradation curve of sulfonamides (a) and phenylurea herbicides (b) over U@H2; Effect of positive ions (c), negative ions (d) and different concentrations of Fulvic acid (e) on the photocatalytic degradation over U@H2, respectively.

Note: In the degradation experiments, 20 mg photocatalyst was added to the solution of sulfonamides (the concentration of sulfamethoxydiazine, sulfapyridine, sulfamethazine, sulfamerazine, sulfadiazine and sulfathiazole 2 mg L^{-1}) and phenylurea (the concentration of metoxuron, chlorotoluron, diuron and isoproturon was 2 mg L^{-1}). 1 mL of the suspension was taken out per 10 min after irradiation. The

residual sulfonamides or phenylurea was analyzed on HPLC-MS/MS and the MRM transitions and other HPLC-MS/MS parameters were described in Table S4. As we can see, the both sulfonamides and phenylurea can be degraded over the U@H2. The rate difference among those substrates may be caused by the electron density and point charge of different molecules.[2,3] In addition, most inorganic ions have no obvious effects on the degradation progress, while Ca^{2+}, Fe^{3+} and $H_2PO_4^-$ may act as competitors for the active species and decrease the degradation rate of TC. The high concentration of fulvic acid (a kind of organic acid widely exists in the environmental soil and water) will also have bad effects on the degradation progress because fulvic acid can absorb light and compete with U@H2 in the water.[4]

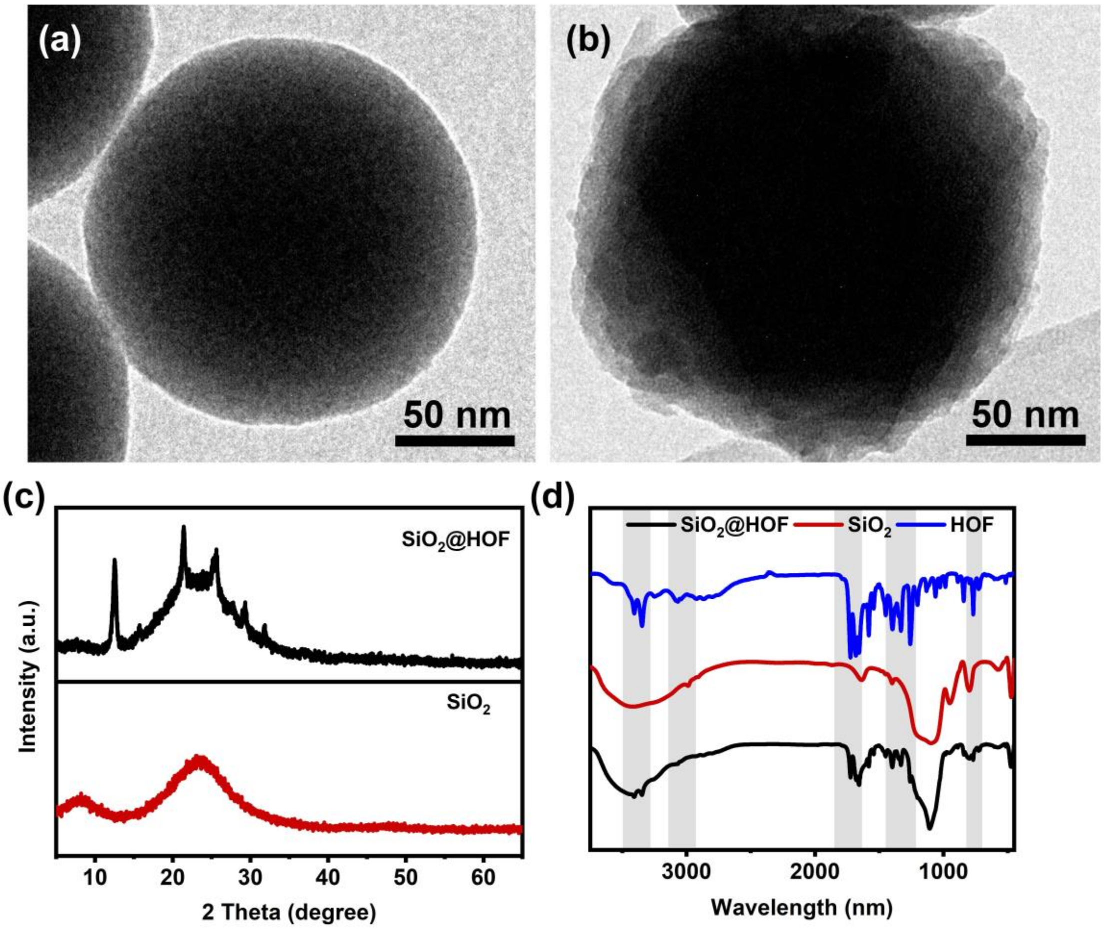

Figure S18. SEM images of SiO_2 (a) and SiO_2@HOF (b); XRD (c) and FT-IR (d) patterns of SiO_2 and SiO_2@HOF.

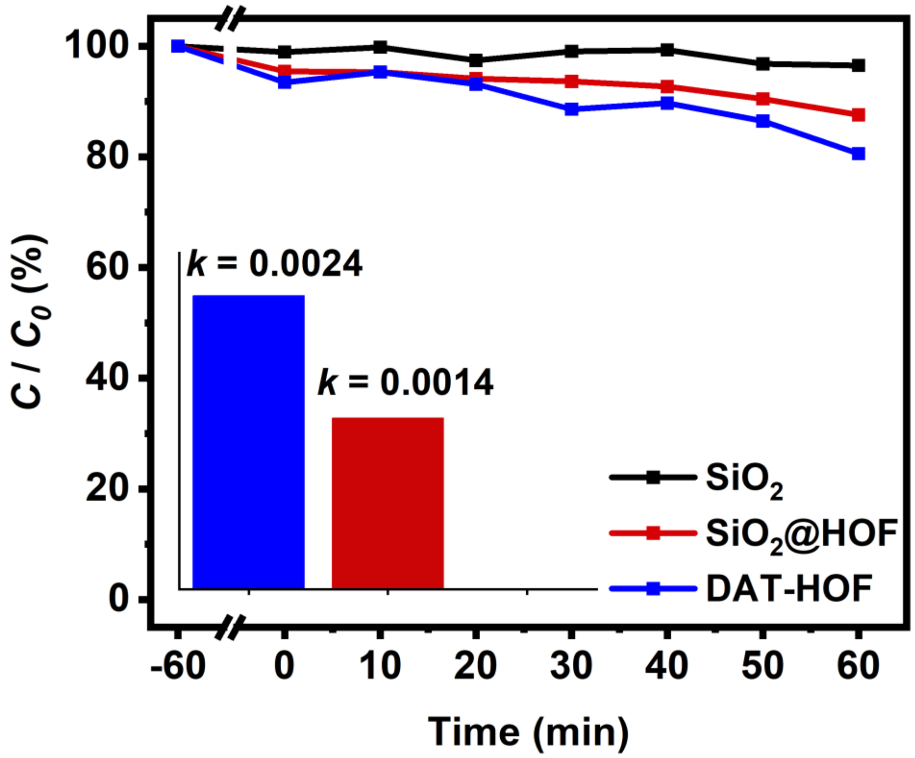

Figure S19. Comparisons of photodegradation efficiencies of TC and the apparent reaction rate constants (inset) in the presence of SiO_2 and SiO_2@HOF.

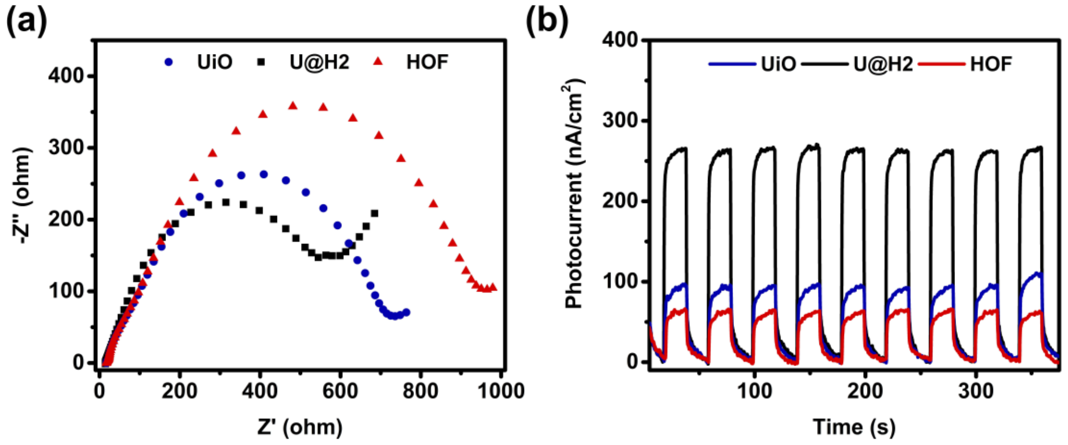

Figure S20. (a) EIS Nyquist curves and (b) Transient photocurrent response of samples.

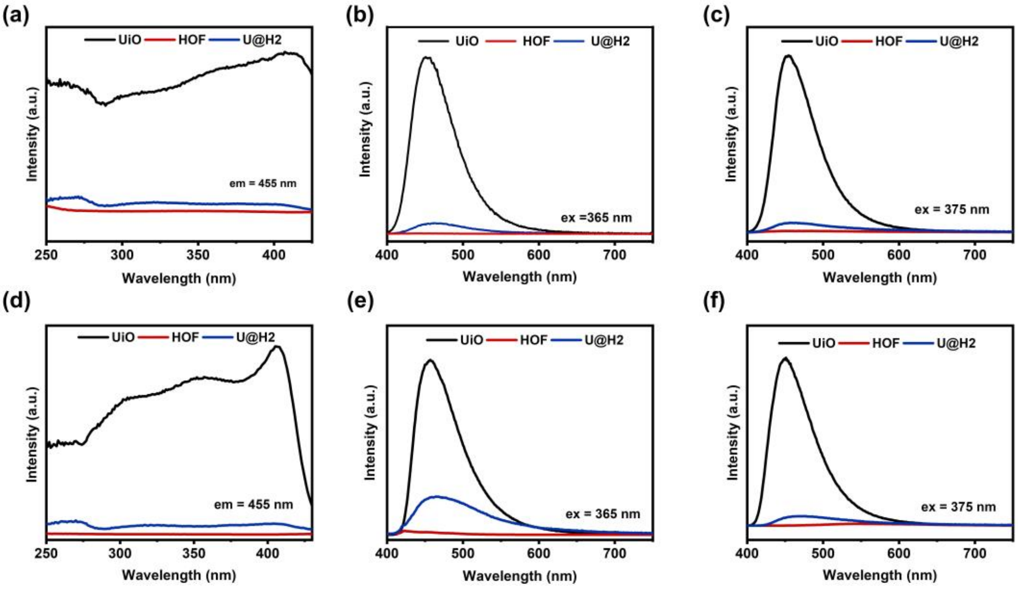

Figure S21. The excitation (em = 455 nm) and emission (ex = 365, 375 nm) spectra of samples in in solid-state (a, b and c) and water suspension (b, c and d).

Note: The PL spectra performed in solid-state was similar with that in suspension, indicating the consistent luminescent behavior in the water suspension.

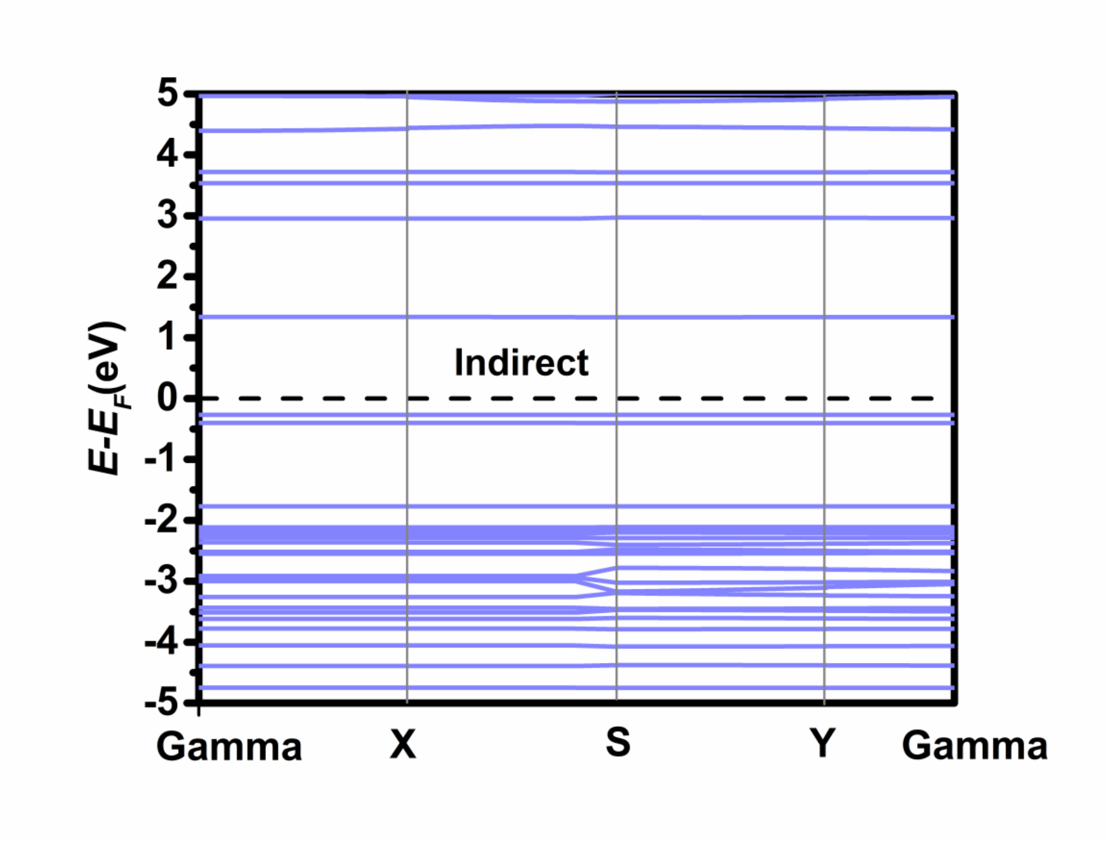

Figure S22. DFT band structure of HOF.

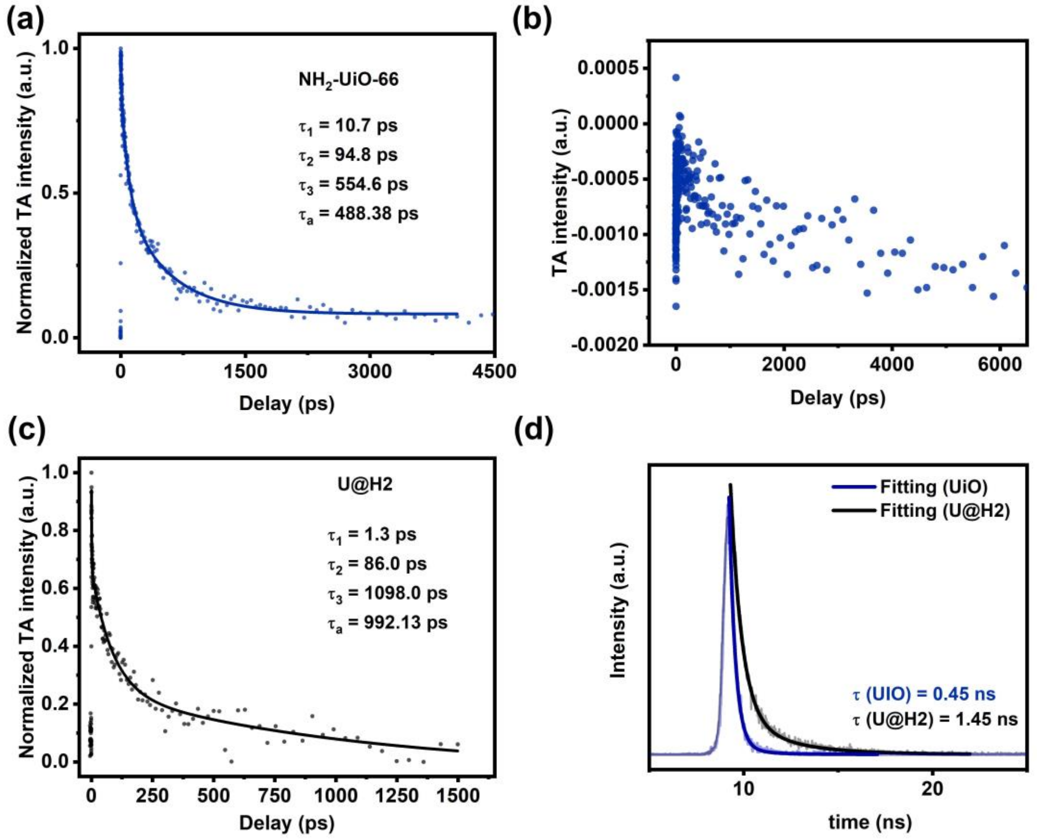

Figure S23. TAS kinetics probed at 650 nm (a) and 500 nm (b) for UiO, (c)TAS kinetics probed at 500 nm for U@H2, (d) FL-RL spectra of UiO and U@H2.

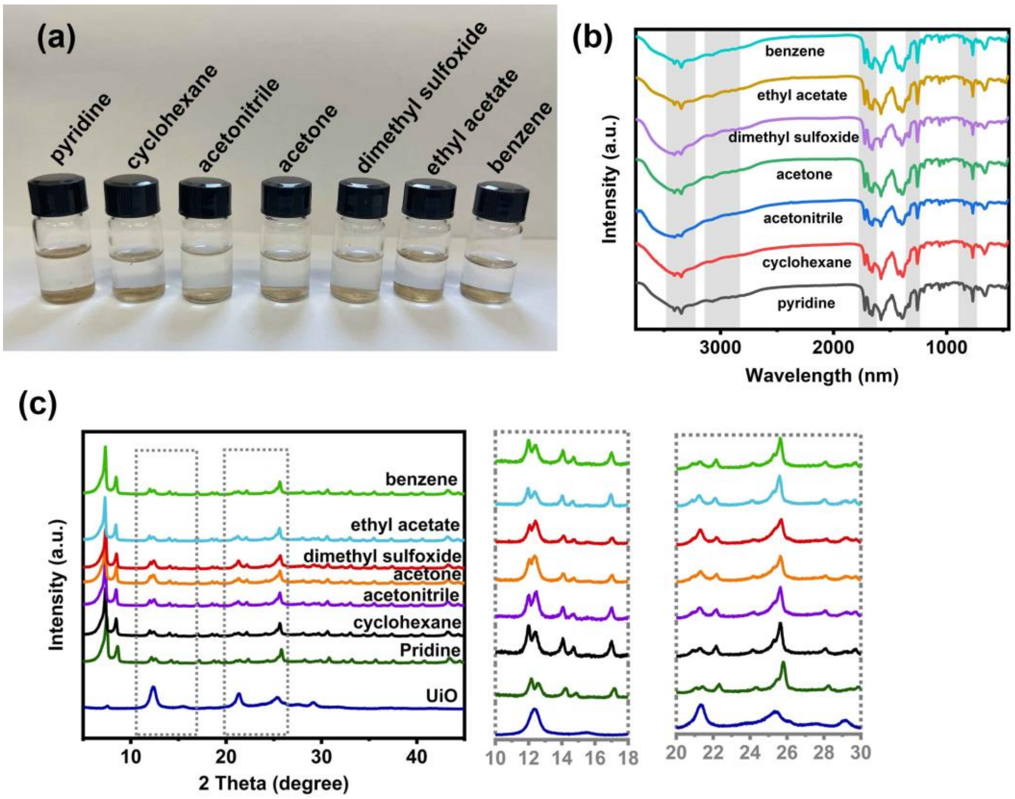

Figure S24. (a) Solubility of U@H2; (b) The FT-IR spectrum of soaked U@H2 in different solvent; (c) The XRD patterns of soaked U@H2 in different solvent.

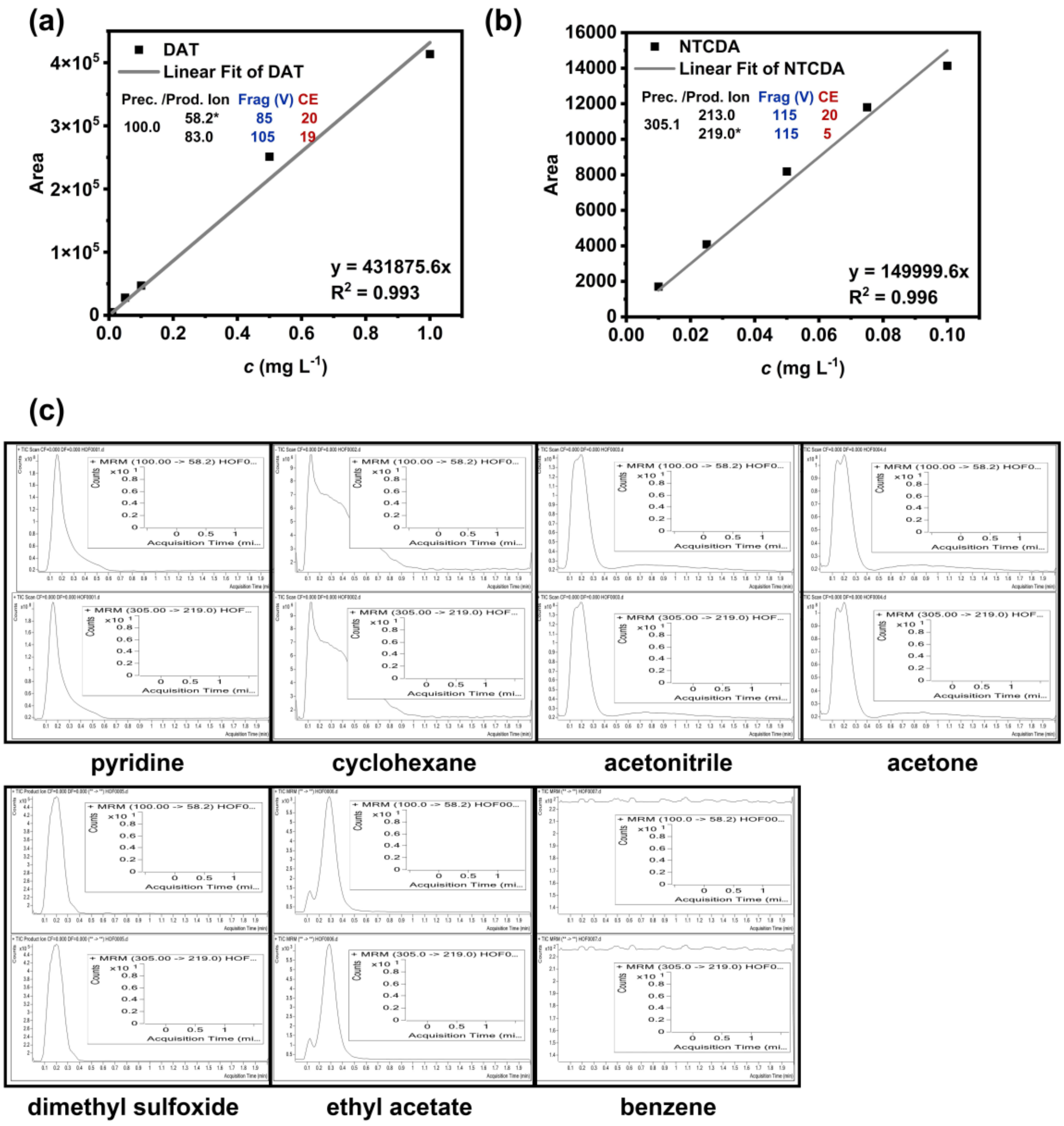

Figure S25. (a, b) The linear regression curves and the HPLC-MS/MS parameters of DAT and NTCDA; (c) The results of HPLC-MS/MS for detecting the filtrate of different U@H2 suspension.

Note: The LOQ (limit of quantitation) values of DAT and NTCDA were both 0.01 mg L^{-1} in the analytical methods. The results of HPLC-MS/MS shows that both the contents of DAT and NTCDA were less than 0.01 mg L^{-1}, which indicates that the monomer of HOF was not destroyed in those solvents.

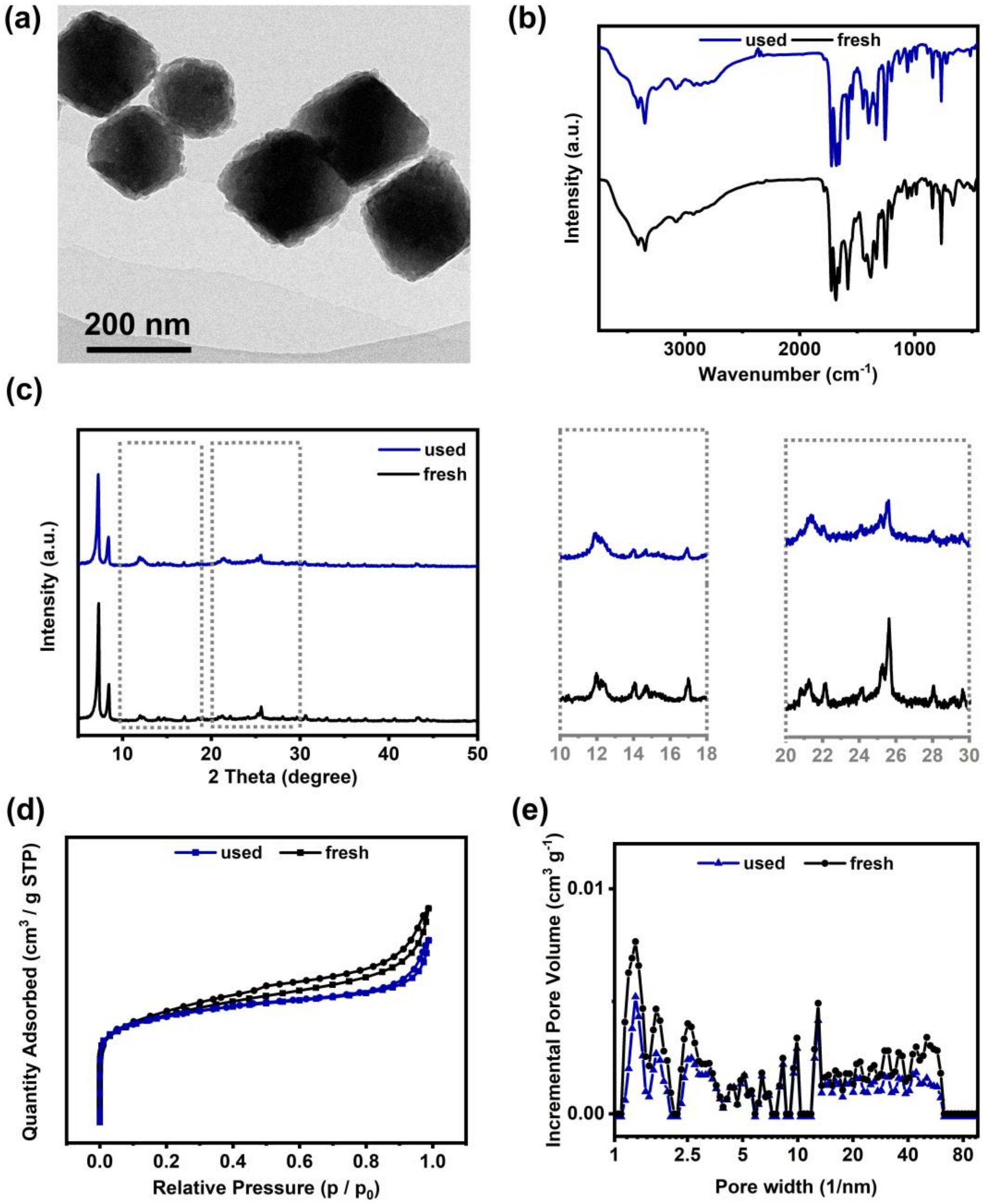

Figure S26. (a) SEM image; (b) FT-IR spectrum; (c) XRD pattern; (d) N_2 adsorption-desorption curve and (e) pore size distribution of UiO after 8 cyclic runs.

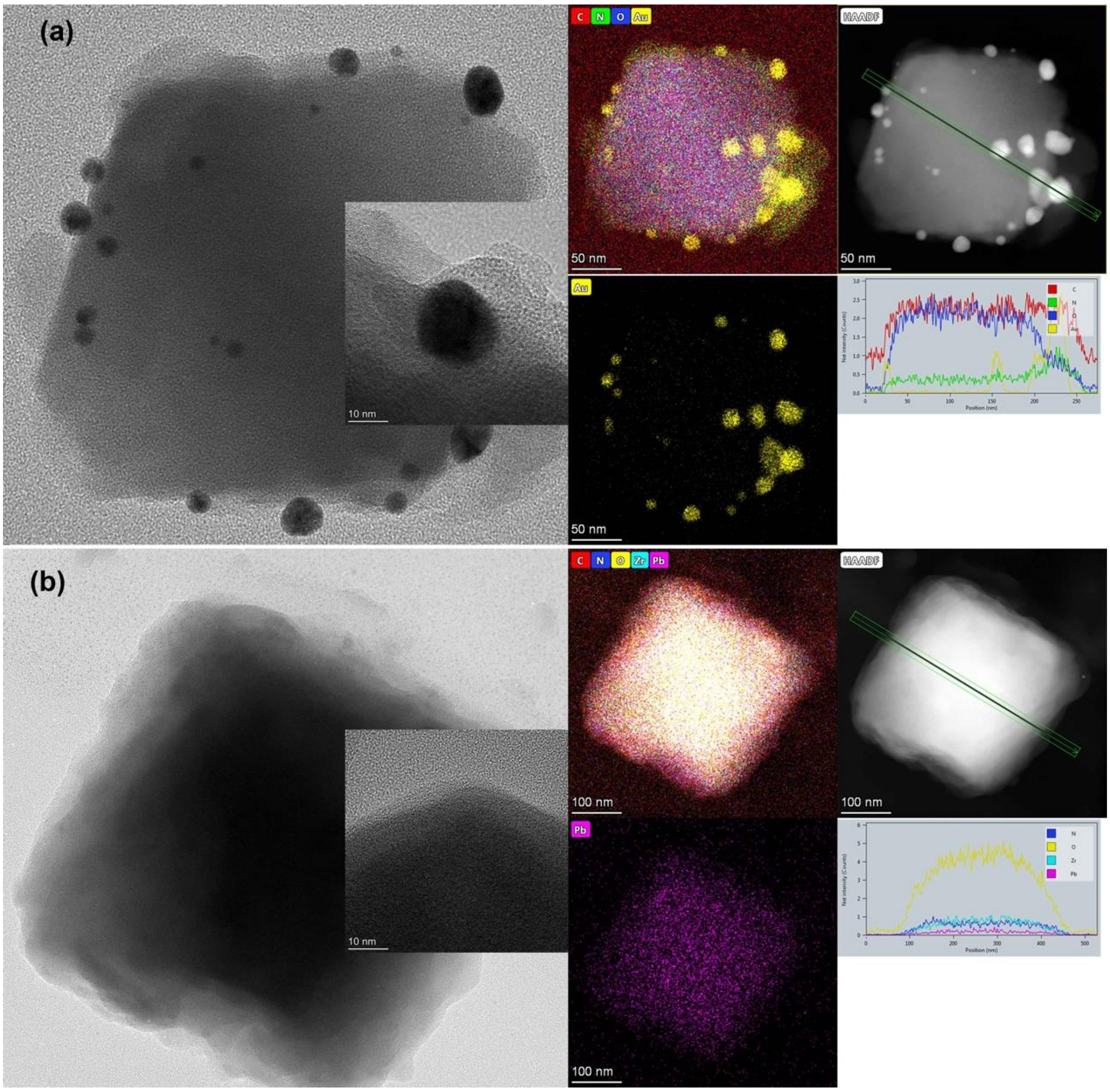

Figure S27. Photodeposition experience of Au (a) and PbO_2 (b).

Note: The photo deposition of Au was reduced from H_2AuCl_4 by acceptance of an electron, and PbO_2 was oxidized from $Pb(NO_3)_2$ by accepting hole. Once U@H2 was irradiated and generated photogenerated electron/hole, the Au^{2+}/Pb^{2+} would be reduced/oxidized into Au/PbO_2 nanoparticles on the surface. As shown in Figure S22, the Au nanoparticles are successfully deposited on the surface of U@H2, while no PbO_2 nanoparticles could be found. It is indicated that the photogenerated electrons were manlily transfer to the surface of U@H2, i.e., the HOF shell. In addition, the Pb elements appeared at the elemental mapping images were mainly concentrated in the UiO core. It is inferred that the photogenerated hole were manlily maintained in the

inner of the U@H2, i.e., the UiO core. There are no obvious coarse particles appear in the images, it probably due to the size limitation of the pore in UiO.

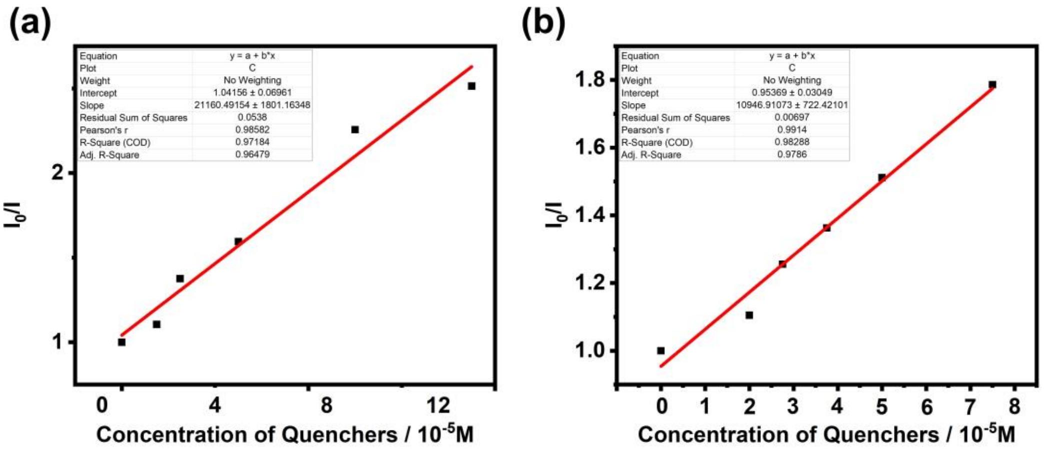

Figure S28. Stern–Volmer quenching experiments of the excited UiO (a) and U@H2 (b) with TC solution.

Note: The Stern–Volmer quenching experiments shows that the excited photocatalysts (UiO and U@H2) can be quenched by TC, where the K_{SV} for UiO is larger than that for U@H2, indicating that the UiO is quenched more easily by TC molecules. It is probably that the bigger specific surface area of UiO causes the more contact with quenchers. Although the excited UiO could collide with TC molecules and causes more charge transfer, which however offer little help for TC photocatalytic degradation for the minor role of electrons during the TC degradation. In contrast, the more quench with TC molecules is might bad for the generation of reactive oxygen species, which affects the degradation process.

Table S1. The yields of samples.

Sample	Yields / mg	Average Yields /mg
UiO	171 (3) [1]; 318 (5); 326 (5); 349 (5); 620 (10); 239 (5); 2400 (32); 2248 (32)	68
DAT-HOF	78; 65; 249 (3); 372 (5);	76
U@H1	214; 208; 220	214
U@H1.5	225	225
U@H2	228; 252; 241; 242; 241; 1195 (5); 720 (3); 1140 (5)	237
U@H2.5	263	263
U@H3	308; 311	310
U@H4	402; 390	396
U/H	257, 260	259
MIL	843(10); 1812 (20)	89
MIL@HOF	227, 236, 220	228

[1] The products were obtained through 3 groups of parallel experiments, and the total yield is 171 mg. The numbers in parentheses are the groups of parallel experiments.

Table S2. The microanalysis data of samples.

Sample	C (Wt.%)	N (Wt.%)	H (Wt.%)	O (Wt.%)	Zr (Wt.%)
DAT-HOF	29.84	49.65	2.462	17.25	-
UiO	4.21	28.77	3.965	25.32	29.28
UiO@HOF	11.34	36.68	3.391	22.19	22.05

Note: The C, N, H and O elements were detected on elements analyzer, and the Zr element was detected on Inductively coupled plasma mass spectrometry (ICP-MS).

Table S3. The fitting results according to the pseudo-first-order kinetic model.

Sample	Fitting curve	k	$k_{avg.}$
U@H1	$y = 0.0127\,x + 0.0362$; $R^2 = 0.9582$	0.0127	0.0142
	$y = 0.0169\,x + 0.0544$; $R^2 = 0.9533$	0.0169	
	$y = 0.0131\,x + 0.0263$; $R^2 = 0.9925$	0.0131	
U@H2	$y = 0.0174\,x + 0.0514$; $R^2 = 0.9853$	0.0174	0.0182
	$y = 0.0184\,x + 0.1180$; $R^2 = 0.9563$	0.0184	
	$y = 0.0187\,x + 0.1448$; $R^2 = 0.9558$	0.0187	
U@H3	$y = 0.0130\,x + 0.0415$; $R^2 = 0.9810$	0.0127	0.0146
	$y = 0.0150\,x + 0.0743$; $R^2 = 0.9254$	0.0127	
	$y = 0.0158\,x + 0.0625$; $R^2 = 0.9595$	0.0127	
U@H4	$y = 0.0085\,x + 0.0020$; $R^2 = 0.9729$	0.0085	0.0108
	$y = 0.0116\,x – 0.0197$; $R^2 = 0.9787$	0.0116	
	$y = 0.0123\,x – 0.0171$; $R^2 = 0.9636$	0.0123	
HOF	$y = 0.0018\,x – 0.0112$; $R^2 = 0.7916$	0.0018	0.0024
	$y = 0.0025\,x – 0.0331$; $R^2 = 0.6424$	0.0025	
	$y = 0.0030\,x – 0.0081$; $R^2 = 0.1248$	0.0030	
UiO	$y = 0.0001\,x – 0.0087$; $R^2 = 0.1827$	0.0001	0.0003
	$y = 0.0004\,x – 0.0249$; $R^2 = 0.2667$	0.0004	
	$y = 0.0005\,x – 0.0362$; $R^2 = 0.9582$	0.0005	
U/H	$y = 0.0015\,x + 0.0124$; $R^2 = 0.6993$	0.0015	0.0013
	$y = 0.0011\,x – 0.0046$; $R^2 = 0.6011$	0.0011	
	$y = 0.0013\,x + 0.0130$; $R^2 = 0.6826$	0.0013	
N-UiO	$y = 0.0007\,x – 0.0137$; $R^2 = 0.6795$	0.0007	0.0011
	$y = 0.0014\,x – 0.0036$; $R^2 = 0.8027$	0.0014	
EDTA-added	$y = 0.0138\,x + 0.0182$; $R^2 = 0.9982$	0.0138	0.0145
	$y = 0.0151\,x + 0.0129$; $R^2 = 0.9949$	0.0151	
	$y = 0.0147\,x + 0.0196$; $R^2 = 0.9922$	0.0147	
$KBrO_3$-added	$y = 0.0148\,x + 0.0774$; $R^2 = 0.9750$	0.0148	0.0159

t-BuOH-added	$y = 0.0170\,x + 0.1266$; $R^2 = 0.9417$	0.0170	0.0159
	$y = 0.0156\,x + 0.0006$; $R^2 = 0.9967$	0.0156	
	$y = 0.0162\,x + 0.0248$; $R^2 = 0.9940$	0.0162	
p-BQ-added	$y = 0.0097\,x + 0.0814$; $R^2 = 0.9357$	0.0097	0.0101
	$y = 0.0105\,x + 0.5060$; $R^2 = 0.9755$	0.0105	
MIL	$y = 0.0001\,x + 0.0433$; $R^2 = 0.0027$	0.0001	0.0001
MIL@HOF	$y = 0.0175\,x – 0.0100$; $R^2 = 0.9917$	0.0175	0.0175

Table S4. MRM transitions and other HPLC-MS/MS parameters of investigated sulfonamides and phenylurea.

Sample	Prec. Ion	Prod. Ion	Frag (V)	CE
Sulfadiazine	251.0	108.3	100	23
		155.8*	105	14
Sulfathiazole	255.3	108.2*	92	25
		155.8	93	13
Sulfapyridine	250.0	156.0*	100	15
		184.0	100	15
Sulfamerazine	265.1	156.0*	100	10
		172.0	100	10
Sulfamethazine	279.2	186.0*	108	10
		124.1	114	23
sulfamethoxydiazine	281.1	156.0*	112	11
		126.0	117	18
Diuron	233.0	72.1*	110	20
		46.3	110	15
Isoproturon	207.2	72.1*	135	15
		46.3	135	15
Metoxuron	229.1	72.1*	110	15
		46.3	110	15
Chlorotoluron	213.0	72.1*	100	20
		46.3	100	15

Table S5. Summary data of time-resolved fluorescence decay of U@H2 and UiO.

Samples	τ_1 (ns)	A_1	τ_2 (ns)	A_2	τ_3 (ns)	A_3	τ_{avg} (ns)
UiO (650 nm)	0.08	10.70	0.38	94.80	0.40	554.60	488.38
U@H2 (650 nm)	0.29	0.45	0.19	12.38	0.46	176.14	171.26
U@H2 (500 nm)	0.00214	1.27	0.00326	86.05	0.00221	1097.98	992.13

References:

1. Wang, N., Cheng, G., Guo, L., Tan, B., Jin, S., Hollow Covalent Triazine Frameworks with Variable Shell Thickness and Morphology. *Adv. Funct. Mater.* 2019, 29, 1904781. https://doi.org/10.1002/adfm.201904781
2. Fengliang Wang, Yiping Feng, Ping Chen, Yingfei Wang, Yuehan Su, Qianxin Zhang, Yongqin Zeng, Zhijie Xie, Haijin Liu, Yang Liu, Wenying Lv, Guoguang Liu, Photocatalytic degradation of fluoroquinolone antibiotics using ordered mesoporous g-C_3N_4 under simulated sunlight irradiation: Kinetics, mechanism, and antibacterial activity elimination, *Appl. Catal. B*, 2018, 227, 114-122, https://doi.org/10.1016/j.apcatb.2018.01.024.
3. Jianli Wang, Meng Yuan, Changsheng Li, Bingjie Zhang, Jianhui Zhu, Xianghong Hao, Huizhe Lu, Yongqiang Ma, One-Step construction of Polyimide/NH_2-UiO-66 heterojunction for enhanced photocatalytic degradation of sulfonamides, *J. Colloid Interf. Sci.*, 2022, 612, 536-549, https://doi.org/10.1016/j.jcis.2021.12.190.
4. Olga M.S. Filipe, Eduarda B.H. Santos, Marta Otero, Elsa A.C. Gonçalves, M. Graça P.M.S. Neves, Photodegradation of metoprolol in the presence of aquatic fulvic acids. Kinetic studies, degradation pathways and role of singlet oxygen, OH radicals and fulvic acids triplet states, *J. Hazard. Mater*, 2020, 385, 121523. https://doi.org/10.1016/j.jhazmat.2019.121523.
